# The effects of parenting on early adolescents’ noncognitive skills: Evidence from a sample of twins in Germany

**DOI:** 10.1177/00016993211051958

**Published:** 2021-11-15

**Authors:** Michael Grätz, Volker Lang, Martin Diewald

**Affiliations:** 27213 University of Lausanne, Switzerland; 7675 Stockholm University, Sweden; 9188 University of Tübingen, Germany; 9167 Bielefeld University, Germany

**Keywords:** adolescents, child development, noncognitive skills, parenting, twins

## Abstract

Many theories in the social sciences assume that parenting affects child development. Previous research mostly supports the notion that parenting affects the skill development of children in early childhood. There are fewer studies testing whether parenting in early adolescence has such an influence. We estimate the effects of parenting on early adolescents’ noncognitive skills using data from the German Twin Family Panel (TwinLife). Specifically, we look at the effects of parenting styles, parental activities, and extracurricular activities on the academic self-concept, motivation, self-esteem, self-efficacy, and locus of control of 10 to 14 years old children. To control for unobserved heterogeneity and reverse causality, we employ twin fixed-effects models combined with longitudinal information. In addition, MZ twin fixed effects models also control for genetic confounding. Our findings provide no support to the notion that parenting styles, parental activities, and extracurricular activities in early adolescence affect the development of children's noncognitive skills. We conclude that our results, in combination with the majority of evidence from previous research, are in line with a model according to which parenting has larger effects on the skill development of children in early childhood than in early adolescence.

## Introduction

Many theories in the social sciences assume that parenting causally affects child development (e.g. Kiernan and Mensah, 2011; Yeung et al., 2002). Research testing this relationship using causal identification strategies has mostly focused on early childhood. This is true for both observational (Del Bono et al., 2016; Fiorini and Keane, 2014; Grätz and Torche, 2016; Hsin and Felfe, 2014; Price and Kalil, 2018) and experimental studies (Chacko et al., 2018; Fryer et al., 2015; Gertler et al., 2014; Lam et al., 2013; Sylva et al., 2008; York et al., 2018). Early childhood is an important period for studying influences of parenting on the skill development of children. However, later periods of childhood and adolescence may be important as well. We estimate the effects of parenting in early adolescence, i.e. when children are 10 to 14 years old, on children's skill development.

The second contribution of our study is that we study heterogeneity in the effects of different dimensions of parenting on skill development. We distinguish between two dimensions of parenting. First, parenting styles are the emotional climates in which parent-child interactions in a family take place reflecting parents’ attempts to control and to socialize their children (Baumrind, 1966, 1991; Chan and Koo, 2011; Cobb-Clark et al., 2019; Darling and Steinberg, 1993; Dooley and Stewart, 2007; Fiorini and Keane, 2014; Spera, 2005). Second, parental activities refer to activities parents engage in to develop children's skills. Previous research often used indicators of parental time use with children and reading to the child (Fiorini and Keane, 2014; Hsin and Felfe, 2014; Leibowitz, 1977; Milkie et al., 2015; Price and Kalil, 2018). Related are extracurricular activities, which are often orchestrated by parents and are an expression of parental cultural capital (Jæger, 2011; Lareau, 2011; Weininger et al., 2015).

We focus on noncognitive skills as a measure of child development because noncognitive skills are important for children's further life chances and are, compared to cognitive skills, longer socially malleable (Heckman et al., 2006). We estimate the effects of parenting styles and parental activities on early adolescents’ noncognitive skills using panel data on a sample of monozygotic (MZ) and dizygotic (DZ) twins aged 10 to 14 from Germany. We implement a research design that combines twin fixed-effects models with longitudinal information. We investigate whether parenting styles, parental activities, extracurricular activities, and ensembles of parenting (combinations of parenting styles and parental or extracurricular activities) in early adolescence influence children's noncognitive skills. We also investigate heterogeneity in the effects of these different dimensions of parenting on skill development by family socioeconomic background.

## Theory

### Does parenting affect child development?

Parenting was proposed as a major channel through which parents affect child development (e.g. Kiernan and Mensah, 2011; Yeung et al., 2002). Empirical support for the importance of parenting comes, among others, from Lareau’s (2011) ethnographic work. In addition, many quantitative studies found that several dimensions of parenting were associated with children's cognitive and noncognitive skills (e.g. Chan and Koo, 2011; Cobb-Clark et al., 2019; Cosconati, 2009; Doepke and Zilibotti, 2017; Ermisch, 2008; Harris et al., 1998; Kaiser et al., 2019; Kiernan and Mensah, 2011; Leibowitz, 1977; Milkie et al., 2015; Thomsen, 2015).

To answer the research question of our study requires, however, to identify the causal effects of parenting on child development. In observational studies, unobserved variables may confound the relationships between parenting styles, parental activities, and children's skills. Possible confounders are genetic variation, child endowments, and early socialization. These factors are often unobserved in observational data.

In addition to unobserved variables, reverse causality can confound the relationship between parenting and child development. Parents may adapt their parenting in response to children's endowments (Becker and Tomes, 1976; Behrman et al., 1982). Empirical results are equivocal but most studies found parents to reinforce initial endowments (Almond and Mazumder, 2013).^1^

**Figure 1.**
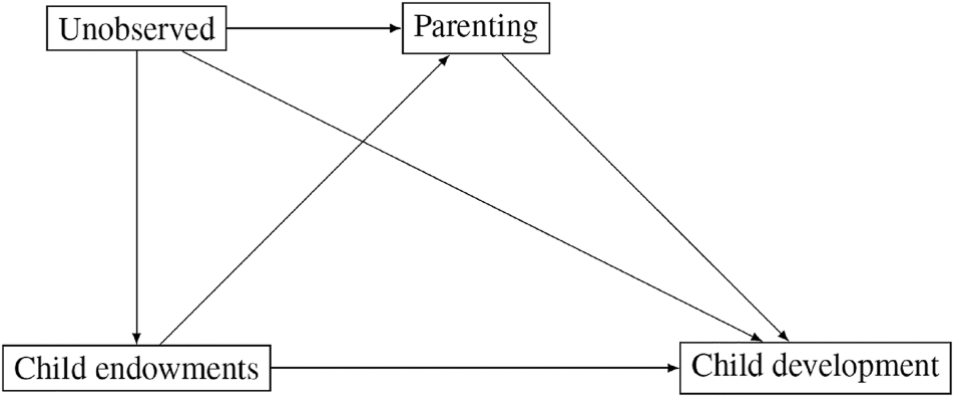
Directed acyclic graph (DAG) of the relationship between child endowments, parenting, and child development.

Figure 1 summarizes the relationships between child endowments, parenting, and child development in a Directed Acyclic Graph (DAG). Child endowments confound the identification of the effect of parenting on child development since parenting is a function of child endowments and child endowments have directs effects on child development. For these reasons, it is necessary to apply a research design that controls for unobserved heterogeneity and reverse causality to estimate the effects of parenting on child development.

Only a small number of studies estimated the causal effects of parenting on children's skills using nationally representative data and research designs that controlled for the influence of unobserved variables. These studies mostly focused on early childhood. Using individual fixed-effects models applied to panel data from the United States, Hsin and Felfe (2014) found that maternal time spent in educational activities, i.e. helping with homework and reading with the child, positively affected children's development of cognitive skills between ages zero and twelve.

Fiorini and Keane (2014) applied the production function approach of Todd and Wolpin (2007) to estimate the effects of parental activities and parenting styles on children's skill development. They compared different specifications (including value added and fixed effects models) and reported results on a cohort from the Longitudinal Study of Australian Children, who were, on average, aged four years and nine months at the first wave, six years and ten months at the second wave, and eight years and ten months at the third wave of data collection. They found parent-child time spent on educational activities to improve children's cognitive but not their noncognitive skills and mother's parenting style to affect children's noncognitive skills.

Del Bono et al. (2016) employed data from the British Millennium Cohort Study and a combination of individual fixed-effects and lagged variable models. They found positive effects of maternal time investments on the development of children's cognitive skills between ages three and seven.

Price and Kalil (2018) used miscarriages to instrument birth order differences in parental reading time to estimate the effects of parental reading on children's reading skills in mid-childhood (age three to nine) in the United States. This method assumed that miscarriages and birth spacing did not affect children's reading skills in other ways than via increasing parental reading time.

Grätz and Torche (2016) used twin fixed-effects models and data on the United States. They found that parental cognitive stimulation around age two did not affect the development of children's cognitive and noncognitive skills between ages two and four.

A related literature employed field experiments to estimate the effects of parenting on children's outcomes. Sylva et al. (2008) conducted a randomized controlled trial in London in which socioeconomically disadvantaged parents were trained to help their five to six years old children with reading. This intervention improved children's reading and writing skills.

Lam et al. (2013) targeted preschoolers, who were, on average, 4.7 years old, in Hong Kong with an intervention that increased parent-child shared book reading. The intervention increased children's performance in vocabulary tests.

Gertler et al. (2014) reported results of an intervention that taught parenting skills in the 1980s to mothers of nine to 24 months old stunted children in Jamaica. The follow-up showed that 20 years later the treated children had higher earnings than the untreated children.

Attanasio et al. (2014) analyzed how increases in parenting affected cognitive skills in Colombia. They found that increased parental stimulation at children's ages 12 to 24 months increased both cognitive skills and language development measured around 1.5 years later.

Chacko et al. (2018) conducted an intervention that fostered father-child shared book reading in a sample of low income families with, on average, 4.6 years old children in the United States. They found that this intervention reduced child problem behavior and improved children's verbal skills.

York et al. (2018) found that a mobile phone-based intervention increased parenting and improved children's literacy skills in a sample of children who were, on average, 4.4 years old.

Fryer et al. (2015) estimated the effects of paying parents to attend parenting programs on four-year-old children's cognitive and noncognitive skills among socioeconomically disadvantaged families in Chicago Heights. They found stronger positive effects on noncognitive than on cognitive skills.

To sum up, the literature applying causal identification strategies found mostly positive effects of parenting on child development. These studies focused on early childhood and not, as we do, on adolescence. Parenting may be more important at younger than at older ages in line with the economic theory of decreasing returns to parenting over the life course due to lesser malleability of the organism (Cunha and Heckman, 2008). However, parenting could also be important in adolescence. This can be the case because adolescence can be a stressful time for the children, as they become more independent. Parenting may help adolescents to deal with the challenges in this part of the life course by avoiding problems which are detrimental for skill development (Larson and Ham, 1993; Milkie et al., 2015; Rudolph and Hammen, 2003). We cannot estimate variation in the impact of parenting on children by age. Rather, we compare our results covering early adolescence to the results of previous research, which focused on early childhood.

### Which aspects of parenting influence child development?

We distinguish between two dimensions of parenting: (1) parenting styles and (2) parental and extracurricular activities. Parenting styles reflect parents’ attempts to control and to socialize their children, creating the emotional climates in which parent-child interactions in a family take place (Baumrind, 1966, 1991; Chan and Koo, 2011; Cobb-Clark et al., 2019; Darling and Steinberg, 1993; Dooley and Stewart, 2007; Fiorini and Keane, 2014; Spera, 2005). We measure parenting styles in one of the possible ways found in the literature (Spera, 2005) based on combinations of parents’ emotional warmth to their children (also called responsiveness or support) and the amount of control parents exercise over their children's behaviors (also called demandingness) (Baumrind, 1991; Huver et al., 2010). Based on these two variables, researchers distinguish up to four parenting styles (Baumrind, 1991; Chan and Koo, 2011; Darling and Steinberg, 1993). First, an authoritative parenting style implies that parents rear their children with a high level of warmth and with a high level of control over children's behaviors. Second, a permissive or indulgent parenting style is brought about by high levels of parental warmth but low levels of parental control. Third, an authoritarian parenting style implies a high level of parental control without parental warmth. Fourth, an unengaged or rejecting-neglecting parenting style is defined by providing neither parental warmth nor parental control. Based on this literature, we expect a combination of high parental warmth and high parental control to be most beneficial for children's development. This is our first hypothesis *H1: A higher combination of parental warmth and control positively affects noncognitive skills*.

Parental activities refer to behaviors that parents engage in with their children and that are supposed to positively influence child development (Fiorini and Keane, 2014; Leibowitz, 1977; Milkie et al., 2015; Price and Kalil, 2018; Thomsen, 2015). In addition, social stratification researchers using cultural capital theory (e.g. Jæger, 2011; Lareau, 2011; Weininger et al., 2015) as well as economists (e.g. Cunha and Heckman, 2008) claimed that extracurricular activities that required attention, self-organization, and concentration fostered the acquisition of noncognitive skills. Therefore, our second hypothesis is *H2: Parental and extracurricular activities positively affect noncognitive skills*.

In addition, we look at the influences of ensembles of parenting, i.e. combinations of parenting styles and parental activities. From the theory of “concerted cultivation” (Lareau, 2011) we derive the hypothesis that ensembles of parenting are particularly important for child development. The idea behind this hypothesis is that what parents think and do may be most beneficial if parental thinking and doing is aligned. We expect a high level of parental or extracurricular activities to be most beneficial for child development if it is combined with a positive parenting style (see also Spera, 2005). This can be formulated as *H3: Ensembles of parenting (combinations of high parental warmth or control with parental or extracurricular activities) positively affect noncognitive skills*.

### Do the effects of parenting on child development vary by family socioeconomic background?

We estimate whether parenting affects children differently in socioeconomically advantaged and disadvantaged families. *We expect parenting to be especially beneficial for children from socioeconomically disadvantaged families (H4).* These children may have a less stimulating home environment and experience more stress in the family (Kiernan and Mensah, 2011). Therefore, they could especially profit from a more positive parenting style or additional parental activities. In line with this hypothesis, field experiments, which often focused on socioeconomically disadvantaged families, found positive effects of parenting on child development (Chacko et al., 2018; Fryer et al., 2015; Gertler et al., 2014; Lam et al., 2013; Sylva et al., 2008; York et al., 2018).

## Data and methods

### Data

We use data from the two waves of the German twin family panel (TwinLife) that are currently available, which were collected in 2014/2015 (wave 1) and in 2016/2017 (wave 2) (Diewald et al., 2018). TwinLife is a probability-based register sample of four cohorts of MZ and same-sex DZ twins and their families living in Germany (Lang and Kottwitz, 2020). In total, data on 4097 twin pairs were collected. Face-to-face interviews were conducted with both of the twins separately and with their parents. This allows us to combine information on parenting from different family members. We use the second youngest of the four birth cohorts, which included 1042 twin pairs in the first wave. 756 of these twin pairs participated in the second wave (Lang et al., 2020). These children were aged 10 to 12 years at the first survey wave and 12 to 14 years at the second wave. We restrict our analyses to this cohort because for these children the data contains comprehensive reports on both parenting and noncognitive skills. We also restrict the sample to children living with both biological parents.

To account for missing information on the explanatory variables we use a full information maximum likelihood algorithm (FIML) based on differences within fully observed twin pairs to estimate the twin fixed-effects models.^2^ We follow the recommendation in the literature and do not include missing values on the dependent variables in the estimation (von Hippel, 2007). Therefore, the sample sizes differ across the different outcomes we analyze due to missing values on the dependent variables. Additional analyses using listwise deletion of twins with missing information on any of the explanatory or dependent variables (presented in Table S2 in the *Online Supplement*) led to very similar results.

### Variables

*Noncognitive Skills.* We analyze six noncognitive skills, which are considered relevant for educational attainment and further life chances (Heckman et al., 2006). First, we analyze children's *academic self-concept*, a self-assessment of their education-related skills. Second, we look at their *intrinsic motivation* to attend school as well as, third, specifically with respect to learning (*learning motivation*). Fourth, we look at children's *self-efficacy* as the degree to which children believe to be able to accomplish goals. Fifth, we measure children's *self-esteem*, i.e. their belief to be a valuable person. Sixth, we analyze children's *locus of control*, their assessment of how much control they have over their life (internal locus) compared to being externally determined (external locus).

Each of these six noncognitive skills is constructed as a sum score based on items that are self-reported by the children on a five-category rating scale in a face-to-face interview conducted during the second survey wave. To measure locus of control, four items were used, while three items were used for each of the other five noncognitive skills. All questions underlying these measures are reported in Table S3 in the *Online Supplement.* The scale reliabilities range from a questionable Cronbach's α of 0.64 for locus of control to a good 0.80 for intrinsic motivation (Table S4 in the *Online Supplement*). Because of the questionable reliabilities of locus of control and academic self-concept (α  =  0.67), the interpretation of results should focus upon the four outcomes measured with good reliability (intrinsic motivation, learning motivation, self-efficacy, and self-esteem).

*Parenting.* We distinguish between two dimensions of parenting: (1) parenting styles and (2) parental and extracurricular activities. With respect to *parenting styles*, we measure *warmth* of the parents as an indicator of emotional support. Furthermore, we measure parent's exercise of behavioral *control*. Both of these measures are constructed using a combination of mother's and father's reports of their parenting styles and children's reports on the parenting styles of both parents. All these reports are based on four analogous items for warmth and three analogous items for control, which were answered on a five-category rating scale in a face-to-face interview conducted at the first wave of the survey. We enter an interaction between parental warmth and parental control in our models to characterize the combination of a high level of parental warmth with a high level of parental control, which should positively affect child development (see H1 above). The reliabilities of the parenting styles scales are good (Table S4 in the *Online Supplement*). Cronbach's α's for parental warmth is 0.85. For parental control, Cronbach's α's is 0.76.^3^

With regard to *parental activities*, we construct a cumulative frequency of how often family members conduct different activities with their children like singing, reading, or visiting exhibitions per month, using five items reported by the children in the first survey wave. In addition, we measure *extracurricular activities* of the children like attending a sports club or a choir to further characterize how often they encounter stimulating developmental opportunities. Extracurricular activities are measured with a cumulative frequency per month using seven items reported by one of the parents in the first survey wave. 82% of the reports are provided by mothers and there are no substantial differences in the frequency of extracurricular activities reported by mothers and fathers. All questions underlying the measures of parenting styles and parental activities are reported in Table S6 in the *Online Supplement*.

*Zygosity.* In our analysis, we distinguish between MZ and same-sex DZ twin pairs in order to control for the influence of genetic variation. The zygosity of twins was determined using a questionnaire and was cross-validated with a saliva test.

*Control Variables.* In order to test the robustness of our results, we add in the final part of our analyses a number of control variables to our twin fixed-effects models. These variables control for the influence of confounding, observed variables that vary within twin pairs. We control for children's IQ and birth weight. To measure IQ we include children's age-adjusted test score in a cultural fair intelligence test (CFT-20 R) taken at the first survey wave. Birth weight is a continuous variable. We also control for noncognitive skills reported in the first survey wave to control for reverse causality, i.e. the effects of noncognitive skills on parenting. The measures of most noncognitive skills we analyze were similarly measured and constructed at the first and second survey waves. Exceptions are self-esteem and locus of control, which were not measured at the first survey wave. We therefore cannot control for these two variables.

Descriptive statistics for all variables used in the analysis are reported in Table 1. We report separate descriptive statistics by zygosity. The comparison of descriptive statistics across zygosity groups shows that the standard deviation within twin pairs (SD_within_) for the noncognitive skills we analyze is about 0.05 to 0.10 sum score-points higher for DZ compared to MZ twins. For most of the variables the standard deviation within twin pairs are about two thirds of the size of the standard deviation between twin pairs (SD_between_) for both zygosity groups. Lower are the standard deviations within twin pairs of the parenting style indicators for both zygosity groups. They are about one third to one fourth of the standard deviation between twin pairs. Even though these findings indicate that there is less variation in parenting within than between twin pairs, they also show that there is variation within twin pairs, even within MZ twin pairs. Given such variation, our research design is applicable. Related, Mönkediek et al. (2020) showed that there was as much variation in parenting styles among twins as there was among non-twin siblings, once the analysis conditioned on age differences.^4^

**Table 1.** Descriptive statistics.

	N_twins_	Mean	SD	SD_within_	SD_between_	Min.	Max.	Twin correlation
*Panel A: Dizygotic twins*								
Academic self-concept	766	3.75	0.57	0.35	0.45	2.00	5.00	0.24
Intrinsic motivation	764	3.41	0.78	0.43	0.65	1.00	5.00	0.40
Learning motivation	758	3.84	0.71	0.43	0.56	1.33	5.00	0.25
Self-efficacy	574	3.88	0.57	0.39	0.41	1.00	5.00	0.05
Self-esteem	660	4.22	0.70	0.44	0.55	1.00	5.00	0.21
Locus of control	656	3.33	0.51	0.32	0.39	1.00	5.00	0.20
Parental warmth	786	4.38	0.42	0.19	0.37	2.50	5.00	0.58
Mother, mother's reports	757	4.50	0.47	0.14	0.45	3.00	5.00	0.83
Father, father's reports	623	4.19	0.53	0.12	0.50	2.50	5.00	0.89
Mother, children's reports	758	4.49	0.57	0.35	0.45	1.00	5.00	0.26
Father, children's reports	757	4.27	0.78	0.43	0.64	1.00	5.00	0.38
Parental control	786	2.77	0.50	0.23	0.44	1.25	4.25	0.57
Mother, mother's reports	752	2.84	0.64	0.21	0.60	1.00	5.00	0.78
Father, father's reports	616	2.82	0.64	0.20	0.60	1.00	4.33	0.80
Mother, children's reports	730	2.71	0.74	0.45	0.59	1.00	5.00	0.26
Father, children's reports	734	2.70	0.81	0.50	0.64	1.00	5.00	0.26
Parental activities	712	13.60	9.04	4.88	7.61	0.00	40.00	0.42
Extracurricular activities	786	5.36	3.28	1.03	3.11	0.00	16.00	0.80
Mother's reports	640	5.37	3.22	1.07	3.04	0.00	16.00	0.76
Father's reports	146	5.30	3.53	0.84	3.43	0.00	16.00	0.88
IQ	740	101.43	16.27	9.25	13.39	58.00	148.00	0.36
Birth weight (in kg)	720	2.50	0.55	0.19	0.51	0.71	4.22	0.75
Academic self-concept, wave 1	756	3.92	0.64	0.37	0.53	2.00	5.00	0.35
Intrinsic motivation, wave 1	770	3.86	0.73	0.43	0.59	1.00	5.00	0.30
Learning motivation, wave 1	726	4.04	0.65	0.41	0.51	1.00	5.00	0.23
Self-efficacy, wave 1	772	3.77	0.64	0.42	0.47	1.33	5.00	0.11
Female	786	0.49	0.50	-	-	0	1	1
Parental tertiary education	786	0.60	0.49	-	-	0	1	1
Parental high occupation (EGP class I and II)	786	0.67	0.47	-	-	0	1	1
*Panel B: Monozygotic twins*								
Academic self-concept	540	3.78	0.61	0.32	0.52	1.33	5.00	0.47
Intrinsic motivation	540	3.48	0.75	0.39	0.64	1.00	5.00	0.46
Learning motivation	534	3.91	0.66	0.34	0.56	2.00	5.00	0.46
Self-efficacy	380	3.91	0.61	0.34	0.50	1.00	5.00	0.37
Self-esteem	462	4.21	0.76	0.42	0.63	1.67	5.00	0.38
Locus of control	440	3.30	0.53	0.31	0.43	1.25	5.00	0.33
Parental warmth	544	4.38	0.40	0.17	0.36	3.00	5.00	0.64
Mother, mother's reports	504	4.52	0.46	0.11	0.45	3.00	5.00	0.89
Father, father's reports	408	4.21	0.58	0.11	0.57	1.75	5.00	0.93
Mother, children's reports	512	4.48	0.55	0.32	0.45	2.25	5.00	0.33
Father, children's reports	504	4.26	0.77	0.36	0.68	1.00	5.00	0.56
Parental control	544	2.75	0.56	0.22	0.51	1.17	4.67	0.68
Mother, mother's reports	504	2.81	0.68	0.17	0.66	1.00	4.67	0.88
Father, father's reports	408	2.77	0.63	0.19	0.60	1.00	4.67	0.82
Mother, children's reports	508	2.74	0.79	0.44	0.66	1.00	5.00	0.40
Father, children's reports	498	2.63	0.86	0.46	0.73	1.00	5.00	0.43
Parental activities	484	12.80	9.08	4.68	7.79	0.00	40.00	0.47
Extracurricular activities	544	5.34	3.54	0.73	3.46	0.00	17.00	0.92
Mother's reports	452	5.29	3.46	0.73	3.38	0.00	16.00	0.90
Father's reports	92	5.59	3.93	0.68	3.87	0.00	17.00	0.94
IQ	508	100.98	16.60	7.24	14.94	59.00	148.00	0.62
Birth weight (in kg)	494	2.37	0.50	0.16	0.47	0.63	4.30	0.79
Academic self-concept, wave 1	532	3.90	0.63	0.28	0.57	1.33	5.00	0.60
Intrinsic motivation, wave 1	530	3.88	0.71	0.35	0.62	1.00	5.00	0.52
Learning motivation, wave 1	496	4.06	0.65	0.36	0.54	1.00	5.00	0.39
Self-efficacy, wave 1	534	3.76	0.63	0.35	0.52	1.67	5.00	0.37
Female	544	0.52	0.50	-	-	0	1	1
Parental tertiary education	544	0.52	0.50	-	-	0	1	1
Parental high occupation (EGP class I and II)	544	0.60	0.49	-	-	0	1	1

*Source*: TwinLife, version 4.0.0 (doi:10.4232/1.13539).

*Note:* The second column reports the number of twin pairs with valid information for each variable.

**Figure 2.**
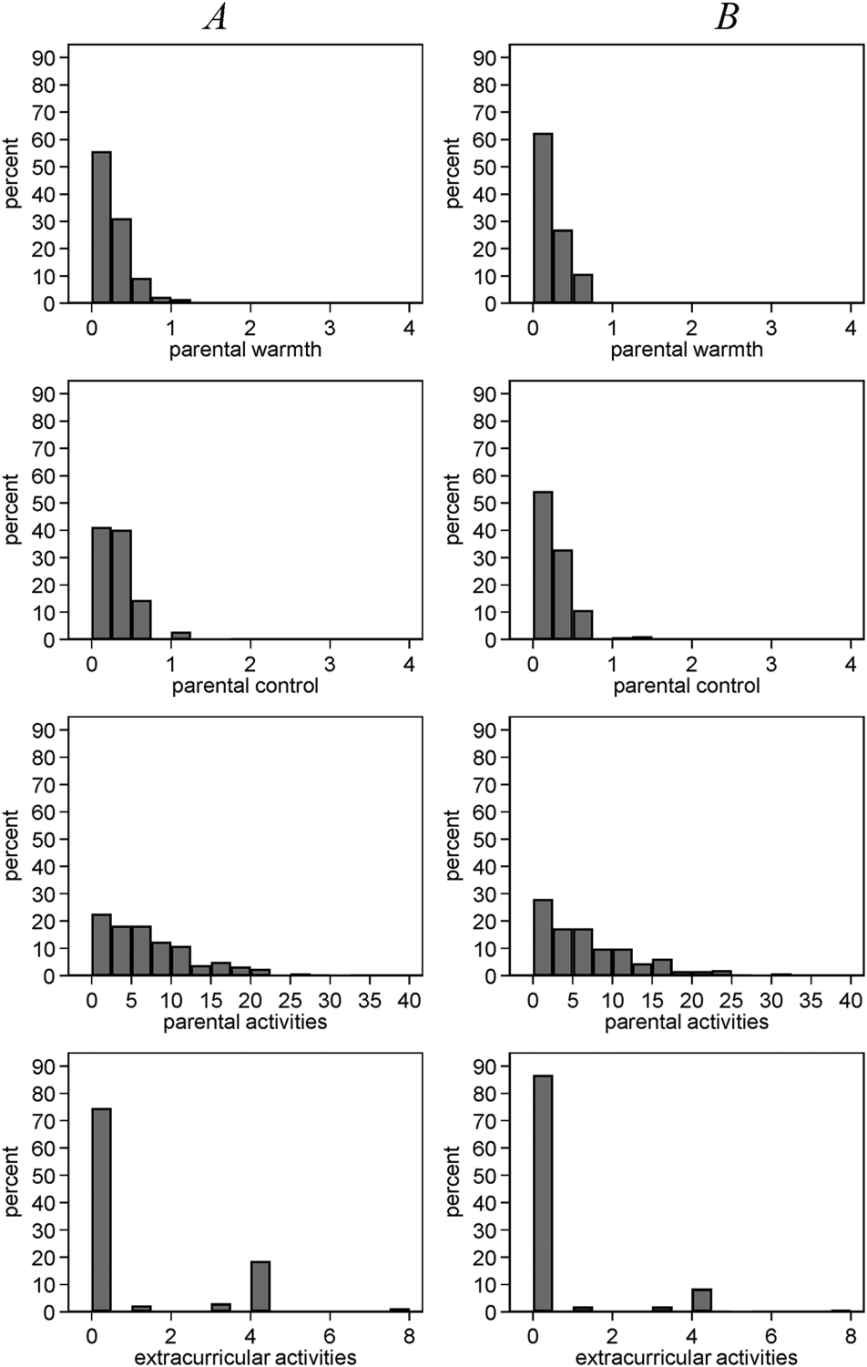
Within-twin pair differences in sum scores of parenting.

*Notes:* Panel A: Dizygotic twins. Panel B: Monozygotic twins. The scales of the sum scores range from 1 to 5. The scales of the cumulative frequencies range from 0 to 40 for parental activities and from 0 to 28 for the extracurricular activities. Since the maximum within-twin pair difference observed for extracurricular activities is 8 only a scale ranging from 0 to 8 is shown for extracurricular activities.

*Source*: TwinLife, version 4.0.0 (doi:10.4232/1.13539).

To look further at how much variation in parenting there is within twin pairs, Figure 2 displays distributions of the within-twin pair-differences in sum scores for our measures of parenting. For all our measures of parenting there is variation within twin pairs. In addition, parents treat DZ and MZ twins differently to about the same degree. These findings should counteract worries that parents do not treat twins differently. Our analysis uses the variation within twin pairs to estimate the effects of parenting on child development. For instance, the graph in the upper left corner shows that for around 60% of twin pairs there is no difference in parental warmth. This finding also implies that 40% of twin pairs show differences in parental warmth. More than 50% of the twin pairs differ in their levels of parental control. About 75% of twin pairs differ in the frequency of activities with their family members. With respect to extracurricular activities, we find the lowest differences between twins (15%). These differences are not large but they are large enough to identify effects of parental warmth on noncognitive skills.^5^

To facilitate the interpretation of our estimates, we z-standardize all variables, i.e. the mean of each variable is set to zero and the standard deviation to one. Therefore, the effects of parenting on children's noncognitive skills can be interpreted in terms of standard deviations. For the z-standardization of the noncognitive skill controls measured at the first survey wave we used the distributions of noncognitive skills measured at the second wave as a reference.

### Analytical strategy

We estimate the effects of parenting on noncognitive skills using twin fixed-effects models. The main strength of these models is that they control for all variables that do not vary between twins, for instance, family socioeconomic background and parental attitudes. They allow us to identify the effects of parenting on noncognitive skills under the assumption that there are no unobserved, confounding variables that vary within twin pairs. Importantly, the MZ twin fixed-effects models also control for genetic differences. Environmental (i.e. non-genetic) variables that vary within twin pairs can, however, lead to biased estimates in MZ twin fixed-effects models if these affect both parenting and noncognitive skills (Kovas et al., 2015). Another limitation of our approach is that twin-fixed effects models use less variation than between-family comparisons, which may hamper the generalizability of results. Whilst we have demonstrated in Figure 2 that variation in parenting exists among twins, this variation is substantively less than between-family variation and could therefore also differ in its effects.

Twin fixed effects models imply that each co-twin is used as a control for a twin. The analysis essentially estimates the effects of twin differences in parenting on twin differences in noncognitive skills. In this manner, we use twin fixed-effects analysis to estimate the effects of parenting on noncognitive skills and therefore interpret our results in this way.

We use measures of noncognitive skills reported at the second survey wave whilst we use measures of parenting reported at the first survey wave. To further account for reverse causality and unobserved variables which affect both parenting and noncognitive skills, we run specifications that include measures of birth weight, cognitive skills, and noncognitive skills measured at the first survey wave as control variables.

Taken together, our research design controls for four sources of unobserved confounding. First, comparing twins allows us to control for unobserved differences between families that arise, for instance, through differences in the parental motivation to foster the development of their children. Second, the comparison of twins allows us to control for changes over time that occur within families and thus, affect comparisons of ordinary siblings. Third, employing the longitudinal information in our data allows us to control for differences between twins that precede our observation of the influence of parenting, for instance child endowments and noncognitive skills of children at an earlier point in time. Fourth, we control for influences of genetic variation by comparing MZ and DZ twins. All models include cluster-robust standard errors at the twin pair-level.

## Results

Table 2 reports twin fixed-effects models of the effects of parenting on noncognitive skills for MZ and DZ twins. These models estimate the effects of parental warmth and parental control, the interaction between these two variables, parental activities, and extracurricular activities, on noncognitive skills. Including the interaction between parental warmth and parental control allows us to test H1.^6^

**Table 2.** Twin fixed-effects models of the effects of parenting on children's noncognitive skills.

	(1)	(2)	(3)	(4)	(5)	(6)
	Academic Self-Concept	Intrinsic Motivation	Learning Motivation	Self-Efficacy	Self-Esteem	Locus of Control
*Panel A: Dizygotic twins*						
Parental warmth	0.10	0.08	0.17*	0.16†	0.19**	0.16†
	(0.07)	(0.07)	(0.07)	(0.09)	(0.06)	(0.08)
Parental control	−0.07	−0.02	0.00	0.03	−0.12†	−0.11
	(0.07)	(0.07)	(0.08)	(0.09)	(0.07)	(0.07)
Parental warmth X Parental control	−0.06	0.06	0.09	0.04	0.08	−0.03
	(0.06)	(0.06)	(0.06)	(0.07)	(0.05)	(0.06)
Parental activities	0.10	0.00	0.05	0.04	−0.04	0.10
	(0.06)	(0.06)	(0.07)	(0.09)	(0.07)	(0.07)
Extracurricular activities	0.02	0.02	−0.07	0.04	−0.04	0.09
	(0.09)	(0.10)	(0.11)	(0.12)	(0.11)	(0.10)
*N* (twins)	766	764	758	574	660	656
*Panel B: Monozygotic twins*						
Parental warmth	0.14†	0.14*	0.06	0.21*	−0.08	−0.04
	(0.08)	(0.07)	(0.07)	(0.10)	(0.10)	(0.10)
Parental control	−0.15†	−0.08	−0.06	0.11	−0.01	0.01
	(0.09)	(0.08)	(0.07)	(0.11)	(0.09)	(0.10)
Parental warmth X Parental control	0.08	−0.09	0.06	0.07	0.02	0.04
	(0.07)	(0.06)	(0.06)	(0.08)	(0.09)	(0.08)
Parental activities	−0.07	−0.02	−0.04	−0.03	0.05	0.07
	(0.07)	(0.06)	(0.07)	(0.07)	(0.08)	(0.09)
Extracurricular activities	0.00	−0.21	−0.02	0.20	−0.01	0.19
	(0.12)	(0.19)	(0.10)	(0.20)	(0.13)	(0.14)
*N* (twins)	540	540	534	380	462	440

*Notes*: All variables are z-standardized. Cluster-robust standard errors in parentheses.

*Source*: TwinLife, version 4.0.0 (doi:10.4232/1.13539).† p < 0.10; * p < 0.05; ** p < 0.01.

We find small positive effects of parental warmth on learning motivation, self-efficacy, self-esteem, and locus of control for DZ twins, and on academic self-concept, intrinsic motivation, and self-efficacy for MZ twins. The strongest effect for DZ twins is found on self-esteem, and for MZ twins on self-efficacy. An increase in parental warmth by one standard deviation increases the self-esteem of DZ twins by 0.19 of a standard deviation as well as the self-efficacy of MZ twins by 0.21 of a standard deviation. These are moderately large effects. However, the pattern is not consistent across the different noncognitive skills we analyze, as for some of these skills weaker effects are found, as well as no effects for some outcomes.

The main effects of parental control are all small in size and statistically insignificant. Contrary to H1, the interaction between parental warmth and parental control has no effects on noncognitive skills. To sum up, we find some evidence that parental warmth is positively related to noncognitive skills but neither parental control nor an authoritative parenting style has effects on noncognitive skills. Furthermore, we find no relevant differences in these effects between DZ and MZ twins.

Furthermore, we find no support for H2: Neither parental activities, nor extracurricular activities, do affect any of the six noncognitive skills we analyze for DZ as well as for MZ twins. Not only are the effects of parental activities on adolescents’ noncognitive skills statistically insignificant but the point estimates are also often substantively close to zero.

The analysis has so far identified the effects of parenting on noncognitive skills in early adolescence using twin-fixed effects models. Next, we test whether our results are robust to including additional control variables. These control variables were measured during the first wave of the survey and therefore prior to noncognitive skills. Table 3 presents the estimates of the effects of parenting on noncognitive skills including the additional control variables for children's IQ, birth weight, and prior noncognitive skills.

**Table 3.** Twin fixed-effects models of the effects of parenting on children's noncognitive skills, controlling for IQ, birth weight, and noncognitive skills at wave 1.

	(1)	(2)	(3)	(4)	(5)	(6)
	Academic Self-Concept	Intrinsic Motivation	Learning Motivation	Self-Efficacy	Self-Esteem	Locus of Control
*Panel A: Dizygotic twins*						
Parental warmth	0.05	−0.05	0.04	0.10	0.15*	0.11
	(0.07)	(0.07)	(0.07)	(0.09)	(0.07)	(0.09)
Parental control	0.00	0.00	0.01	0.11	−0.10	−0.09
	(0.06)	(0.06)	(0.08)	(0.08)	(0.08)	(0.07)
Parental warmth X Parental control	−0.08	0.03	0.06	0.04	0.08	−0.04
	(0.05)	(0.05)	(0.06)	(0.07)	(0.06)	(0.06)
Parental activities	0.05	−0.01	0.03	−0.03	−0.06	0.09
	(0.06)	(0.06)	(0.07)	(0.08)	(0.07)	(0.07)
Extracurricular activities	0.05	0.01	−0.06	0.10	−0.01	0.09
	(0.08)	(0.09)	(0.11)	(0.11)	(0.11)	(0.11)
*N* (twins)	766	764	758	574	660	656
*Panel B: Monozygotic twins*						
Parental warmth	0.13†	0.11†	0.02	0.18†	−0.09	−0.08
	(0.08)	(0.07)	(0.07)	(0.10)	(0.10)	(0.11)
Parental control	−0.14†	−0.05	−0.03	0.16	0.00	0.06
	(0.08)	(0.08)	(0.07)	(0.11)	(0.09)	(0.10)
Parental warmth X Parental control	0.08	−0.10	0.07	0.12	0.02	0.04
	(0.07)	(0.07)	(0.06)	(0.08)	(0.08)	(0.07)
Parental activities	−0.13*	−0.07	−0.10	−0.16*	0.04	0.04
	(0.07)	(0.07)	(0.07)	(0.08)	(0.09)	(0.09)
Extracurricular activities	−0.06	−0.25	−0.05	0.26	−0.04	0.12
	(0.10)	(0.19)	(0.10)	(0.19)	(0.14)	(0.14)
*N* (twins)	540	540	534	380	462	440

*Notes*: All variables are z-standardized. Cluster-robust standard errors in parentheses. All models control for IQ, birth weight, academic self-concept at wave 1, intrinsic motivation at wave 1, learning motivation at wave 1, and self-efficacy at wave 1 (controls not shown).

*Source*: TwinLife, version 4.0.0 (doi:10.4232/1.13539).† p < 0.10; * p < 0.05; ** p < 0.01.

Controlling for IQ, birth weight, and prior noncognitive skills affects our estimates of the effects of parenting on noncognitive skills in early adolescence. The main effects of parental warmth found in twin-fixed effects models without these variables reported in Table 3 are reduced in the models that include the additional control variables. The only remaining statistically significant estimate in Table 3 is the positive effect of parental warmth on the self-esteem of DZ twins. However, since we cannot control for self-esteem at wave 1 with our data, the causal interpretation of this effect is very doubtful. Overall, we can therefore not rule out that parental warmth affects this noncognitive skill in our sample of DZ twins. Nevertheless, overall, our results suggest that parenting styles do not affect noncognitive skills in early adolescence (contrary to H1).

**Table 4.** Twin fixed-effects models of the effects of parenting on children's noncognitive skills, ensembles of parenting, controlling for IQ, birth weight, and noncognitive skills at wave 1.

	(1)	(2)	(3)	(4)	(5)	(6)
	Academic Self-Concept	Intrinsic Motivation	Learning Motivation	Self-Efficacy	Self-Esteem	Locus of Control
*Panel A: Dizygotic twins*						
Parental warmth	0.05	−0.07	0.03	0.06	0.15*	0.10
	(0.07)	(0.07)	(0.07)	(0.10)	(0.07)	(0.09)
Parental control	0.01	0.02	0.02	0.10	−0.13	−0.07
	(0.06)	(0.06)	(0.08)	(0.08)	(0.08)	(0.07)
Parental warmth X Parental control	−0.08	0.02	0.04	0.03	0.06	−0.03
	(0.06)	(0.05)	(0.06)	(0.06)	(0.06)	(0.06)
Parental activities	0.04	−0.01	0.02	−0.03	−0.07	0.08
	(0.06)	(0.06)	(0.06)	(0.08)	(0.08)	(0.06)
Parental warmth X Parental activities	0.03	−0.03	−0.03	0.00	0.00	0.04
	(0.06)	(0.04)	(0.06)	(0.07)	(0.05)	(0.05)
Parental control X Parental activities	0.00	−0.07	0.01	−0.06	0.10†	−0.08
	(0.05)	(0.05)	(0.05)	(0.06)	(0.06)	(0.06)
Extracurricular activities	0.06	0.02	−0.04	0.12	0.01	0.09
	(0.09)	(0.09)	(0.11)	(0.11)	(0.11)	(0.11)
Parental warmth X Extracurricular activities	0.02	0.06	0.01	0.13†	0.04	0.04
	(0.05)	(0.04)	(0.05)	(0.07)	(0.04)	(0.07)
Parental control X Extracurricular activities	0.05	0.06	0.15*	−0.03	−0.01	−0.01
	(0.06)	(0.05)	(0.07)	(0.07)	(0.07)	(0.07)
*N* (twins)	766	764	758	574	660	656
*Panel B: Monozygotic twins*						
Parental warmth	0.12	0.09	0.02	0.17†	−0.07	−0.05
	(0.08)	(0.07)	(0.07)	(0.09)	(0.10)	(0.11)
Parental control	−0.15†	−0.01	−0.03	0.19†	0.02	0.06
	(0.08)	(0.08)	(0.07)	(0.11)	(0.09)	(0.11)
Parental warmth X Parental control	0.09	−0.10	0.07	0.12	0.01	0.03
	(0.07)	(0.07)	(0.06)	(0.08)	(0.08)	(0.07)
Parental activities	−0.13*	−0.05	−0.10	−0.16*	0.03	0.04
	(0.07)	(0.06)	(0.07)	(0.08)	(0.08)	(0.09)
Parental warmth X Parental activities	0.10†	−0.05	0.03	0.00	−0.02	−0.05
	(0.06)	(0.05)	(0.06)	(0.05)	(0.06)	(0.06)
Parental control X Parental activities	0.04	−0.01	0.01	−0.08	−0.13†	−0.04
	(0.06)	(0.06)	(0.06)	(0.08)	(0.07)	(0.08)
Extracurricular activities	−0.03	−0.22	−0.02	0.18	−0.08	0.13
	(0.11)	(0.18)	(0.10)	(0.18)	(0.14)	(0.16)
Parental warmth X Extracurricular activities	−0.08	−0.04	0.05	−0.14	0.11	0.15
	(0.08)	(0.06)	(0.07)	(0.10)	(0.09)	(0.10)
Parental control X Extracurricular activities	0.02	0.15*	0.07	−0.06	0.00	0.00
	(0.07)	(0.07)	(0.06)	(0.07)	(0.08)	(0.08)
*N* (twins)	540	540	534	380	462	440

*Notes*: All variables are z-standardized. Cluster-robust standard errors in parentheses. All models control for IQ, birth weight, academic self-concept at wave 1, intrinsic motivation at wave 1, learning motivation at wave 1, and self-efficacy at wave 1 (controls not shown).

*Source*: TwinLife, version 4.0.0 (doi:10.4232/1.13539).

† p < 0.10; * p < 0.05; ** p < 0.01.

In addition, after adding all control variables, we find a substantially small but statistically significant negative effect of parental activities on the academic self-concept and self-efficacy of MZ twins. Since these small effects are only found using the models with the additional controls variables and only for MZ twins, these findings are not robust The effects point also in another direction than expected in H2, i.e., the findings suggest that more stimulation provided by the parents hinders child development. In addition, we find no effects of parental and extracurricular activities on all other noncognitive skills. Overall, the models including the more extensive set of control variables reported in Table 4 therefore largely demonstrate that, contrary to H2, parental and extracurricular activities do not affect noncognitive skills in early adolescence.

A concern with the models reported in Table 3 is that entering measures of parenting styles and parental as well as extracurricular activities in the same models may lead to multicollinearity and overcontrol bias, as the causal relationships between these variables are unclear. Table S7 in the *Online Supplement* displays the correlations between the different dimensions of parenting. We find weak positive correlations between parental activities and parental warmth as well as between parental activities and extracurricular activities, and a weak negative correlation between parental activities and parental control. We therefore conclude that multicollinearity between the different dimensions of parenting is unlikely to affect our results. To address the concern of overcontrol bias, we tested the robustness of our results using specifications that entered the different indicators of parenting into separate models. These models are reported in Tables S10 to S15 in the *Online Supplement* and produce virtually identical estimates to those reported in Table 4.

In the next step in our analyses, we test H3 by assessing the influence of specific combinations of parenting styles, parental activities, and extracurricular activities by looking at the effects of the interactions between these variables. These results are shown in Table 4.

We find for a large majority of interactions no substantively large and statistically significant effects. There are only two exceptions. First, we find a statistically significant interaction between extracurricular activities and parental control on learning motivation for DZ twins. The effect is moderately large in size (0.15) and is not reproduced for MZ twins. In addition, the interaction is very inconsistent across outcomes. The estimates for different outcomes indicate positive, negative, and zero effects. Second, we find a statistically significant and moderately large (0.15) interaction between extracurricular activities and parental control for the intrinsic motivation of MZ twins. This finding is not reproduced for DZ twins. What is more, this result seems again specific to one out of six noncognitive skills as for none of the other outcomes such a large interaction effect is found. Both interaction effects are also neither statistically significant nor substantively large in the models that do not include the control variables from the first survey wave (these models are reported in Table S16 in the *Online Supplement*).

In sum, our results show that, contrary to H3, specific combinations of parenting styles, parental activities, and extracurricular activities in early adolescence do not influence the development of noncognitive skills.

### Socioeconomic differences in the effects of parenting on children's noncognitive skills

We estimate socioeconomic differences in the effects of parenting on noncognitive skills in early adolescence by including interactions between parenting and parental tertiary education (ISCED 5/6, using the parent with the highest level of education). These models are reported in Table 5. As a robustness check, we estimated models including interactions with a high level of parental occupation. We defined as a high level of parental occupation the upper two classes (I and II) of the Erikson-Goldthorpe-Portocarero (EGP) class schema (Erikson and Goldthorpe, 1992). Again, we used the parent with the highest level of occupation in the EGP class schema. These models are reported in Table S17 in the *Online Supplement*.

**Table 5.** Twin fixed-effects models of the effects of parenting on children's noncognitive skills, interactions with parental education, controlling for IQ, birth weight, and noncognitive skills at wave 1.

	(1)	(2)	(3)	(4)	(5)	(6)
	Academic Self-Concept	Intrinsic Motivation	Learning Motivation	Self-Efficacy	Self-Esteem	Locus of Control
*Panel A: Dizygotic twins*						
Parental warmth	0.07	−0.18*	−0.02	−0.03	0.15	0.12
	(0.09)	(0.09)	(0.10)	(0.15)	(0.09)	(0.13)
Parental control	−0.02	0.02	−0.03	0.13	−0.16	−0.12
	(0.09)	(0.10)	(0.10)	(0.15)	(0.12)	(0.11)
Parental warmth X Parental control	−0.08	0.02	0.05	0.04	0.07	−0.04
	(0.05)	(0.05)	(0.06)	(0.07)	(0.06)	(0.06)
Parental activities	0.06	−0.10	−0.07	−0.07	−0.11	0.16
	(0.10)	(0.10)	(0.10)	(0.12)	(0.10)	(0.11)
Extracurricular activities	0.04	0.07	−0.06	0.20	0.15	0.03
	(0.12)	(0.16)	(0.16)	(0.19)	(0.18)	(0.17)
Parental warmth X Parental tertiary education	−0.04	0.24*	0.09	0.22	0.02	0.02
	(0.14)	(0.12)	(0.14)	(0.18)	(0.13)	(0.17)
Parental control X Parental tertiary education	0.03	−0.02	0.09	−0.02	0.08	0.04
	(0.13)	(0.12)	(0.15)	(0.18)	(0.15)	(0.15)
Parental warmth X Parental control X Parental tertiary education	−0.02	−0.01	0.14†	0.01	−0.05	−0.14*
	(0.06)	(0.05)	(0.08)	(0.06)	(0.06)	(0.06)
Parental activities X Parental tertiary education	−0.02	0.15	0.18	0.04	0.09	−0.15
	(0.12)	(0.12)	(0.13)	(0.16)	(0.14)	(0.13)
Extracurricular activities X Parental tertiary education	0.02	−0.13	−0.02	−0.18	−0.24	0.12
	(0.17)	(0.20)	(0.22)	(0.24)	(0.23)	(0.22)
*N* (twins)	766	764	758	574	660	656
*Panel B: Monozygotic twins*						
Parental warmth	0.15	0.11	−0.02	0.29†	−0.04	−0.23†
	(0.11)	(0.09)	(0.10)	(0.16)	(0.15)	(0.14)
Parental control	−0.09	−0.10	−0.01	−0.05	−0.02	0.17
	(0.13)	(0.12)	(0.09)	(0.19)	(0.12)	(0.15)
Parental warmth X Parental control	0.09	−0.11†	0.08	0.10	0.01	0.06
	(0.06)	(0.06)	(0.06)	(0.07)	(0.09)	(0.08)
Parental activities	−0.20†	−0.04	−0.22*	−0.17	−0.02	−0.16
	(0.10)	(0.11)	(0.09)	(0.12)	(0.13)	(0.12)
Extracurricular activities	−0.01	−0.59*	−0.06	0.34	−0.05	−0.35
	(0.17)	(0.27)	(0.17)	(0.29)	(0.22)	(0.23)
Parental warmth X Parental tertiary education	−0.07	−0.01	0.07	−0.11	−0.10	0.26
	(0.15)	(0.14)	(0.14)	(0.21)	(0.20)	(0.20)
Parental control X Parental tertiary education	−0.14	0.09	−0.02	0.30	0.02	−0.29
	(0.15)	(0.15)	(0.15)	(0.22)	(0.19)	(0.20)
Parental warmth X Parental control X Parental tertiary education	−0.05	−0.02	0.02	−0.11	−0.06	−0.17*
	(0.05)	(0.05)	(0.07)	(0.07)	(0.07)	(0.08)
Parental activities X Parental tertiary education	0.13	−0.05	0.20	0.05	0.12	0.36*
	(0.13)	(0.12)	(0.13)	(0.14)	(0.16)	(0.16)
Extracurricular activities X Parental tertiary education	−0.11	0.70*	−0.03	−0.14	0.00	0.65*
	(0.21)	(0.34)	(0.21)	(0.39)	(0.27)	(0.28)
*N* (twins)	540	540	534	380	462	440

*Notes*: All variables (apart from parental tertiary education) are z-standardized. Cluster-robust standard errors in parentheses. All models control for IQ, birth weight, academic self-concept at wave 1, intrinsic motivation at wave 1, learning motivation at wave 1, and self-efficacy at wave 1 (controls not shown).

*Source*: TwinLife, version 4.0.0 (doi:10.4232/1.13539).

† p < 0.10; * p < 0.05; ** p < 0.01.

We find a statistically significant negative effect of parental warmth on intrinsic motivation for DZ twins only in families in which no parent has tertiary education. However, there are no socioeconomic differences in the effects of parental warmth on the other five noncognitive skills. In addition, the interaction between parental warmth and parental tertiary education for MZ twins is zero. Moreover, there is a significant negative effect of the interaction between parental warmth, parental control, and parental tertiary education on the locus of control for DZ and MZ twins. For both zygosity groups, these effects are, however, small. In addition, the robustness check using parental occupation does not reproduce any of these findings.

Only for MZ but not for DZ twins, we find a negative influence of parental activities on learning motivation in families without a tertiary educated parent. In addition, we find a positive effect of extracurricular activities on intrinsic motivation only in families with a tertiary educated parent. These socioeconomic differences are also found in the robustness check using parental occupation (see Table S17 in the *Online Supplement*). Furthermore, we find positive effects of extracurricular activities and parental activities on locus of control for MZ twins in tertiary educated families (but not for DZ twins). These last two results are, however, not reproduced in the analyses using parental occupation. In addition, there are no socioeconomic differences in the effects of parental and extracurricular activities on the other three noncognitive skills, neither for MZ nor for DZ twins.

We conclude that, overall, the effects of parenting on noncognitive skills do largely not vary by family socioeconomic status. Our analysis therefore provides no support to H4, which expected stronger effects of parenting styles and parental activities in socioeconomically disadvantaged families.

### Robustness checks

A concern with our analysis may be that we could have not enough statistical power to detect the potential small effects parenting may have on noncognitive skills in early adolescence. To increase statistical power, we estimated models that combined the DZ and MZ twin panels. We report these models in Table S18 in the *Online Supplement*. These models find also no effects of parenting on noncognitive skills.

In addition, we tested for gender differences in the effects of parenting on noncognitive skills by including interactions between parenting and child gender. These models are reported in Table S19 in the *Online Supplement*. We do find a significant positive effect of parental warmth on two out of six noncognitive skills for parental warmth (learning motivation and locus of control) for DZ twin boys. For girls, these effects are zero. The effects of parental control, parental activities, and extracurricular activities do not differ by child gender for DZ twins. For MZ twin boys, we observe no effects of parenting on noncognitive skills. The only gender difference we find is a negative interaction between extracurricular activities and being female on one out of six noncognitive skills (intrinsic motivation). Other than that, we find no statistically significant differences in the effects of parenting on noncognitive skills between boys and girls. Thus, we conclude that there are largely no gender differences in the effects of parenting on noncognitive skills, if at all, effects of parenting may be stronger for boys than for girls.

## Discussion and conclusion

Parenting is widely believed to affect children's skill development. However, much empirical evidence used to support this claim does not allow researchers to identify causal effects, especially with respect to parenting in adolescence. We used twin fixed-effects models to control for all stable characteristics that varied between twin pairs and utilized a longitudinal design to control for reverse causality to estimate the effects of parenting in early adolescence on six different noncognitive skills. In addition, we compared the effects of parenting styles, parental activities, and extracurricular activities on children's noncognitive skills.

Our findings provide only limited support to the notion that parenting in early adolescence affects the skill development of children. We found some positive associations between parental warmth and several noncognitive skills, but these disappeared mostly once we controlled for earlier noncognitive skills. Our analysis therefore suggests that parenting styles at ages 10 to 14, to a large degree, do not affect children's noncognitive skills. This finding holds for both socioeconomically advantaged and disadvantaged families. As far as there are any effects, they are limited to self-esteem and are concentrated among boys.

In addition, we found that parental and extracurricular activities in early adolescence did not affect children's noncognitive skills. This finding suggests that the causal effects of these activities, found by Attanasio et al. (2014), Gertler et al. (2014), and Price and Kalil (2018), may be limited to early childhood. Thus, our findings, in combination with results from this research, support the notion of a negative age gradient in the impact of parental activities on children's skills. Such a pattern is in line with research claiming that the social environment matters more in early childhood than in adolescence (Cunha and Heckman, 2008).

A limitation of our study is that our research design by definition only exploits variation in parenting within families (Boardman and Fletcher, 2015). This is the trade-off for having much stricter control of third variable confounders (which is the main strength of our research design). Between-family variation in parenting may have stronger effects on child development than within-family variation. Whilst we cannot rule out this possibility, there is also no evidence using causal identification strategies and nationally representative data for between-family variation that would provide empirical support to such a claim, especially for parenting in early adolescence.

The estimates reported in our analysis can also be interpreted as causal effects only under strong assumptions. The causal interpretation of twin fixed effects models assumes that any differences between twins in parenting are not due to unobserved variables, which also affect the outcome of interest (Boardman and Fletcher, 2015). This assumption is relaxed in the models which include measures of noncognitive skills from the first wave as controls. The causal interpretation of these models assumes that there are no unobserved variables which affect parenting and which do not affect differences in noncognitive skills at wave 1 but only noncognitive skills at wave 2.

Some researchers have argued that using twins may result in the amplification of measurement error (Boardman and Fletcher, 2015; Frisell et al., 2012). We have, however, demonstrated in the power analysis reported in note 6 that there is enough statistical power to find effects. In addition, we focused in our interpretation of results both on statistical estimates as well as on the substantive size and the direction of the effects (Bernardi et al., 2017). Finally, we find statistically significant differences in the models which do not include measures of noncognitive skills from wave 1. Therefore, the fact that we find no effects of parenting on noncognitive skills once we control for noncognitive skills from wave 1 is not due to amplified measurement error in the twin fixed effects models.

Two of our outcomes – academic self-concept and locus of control – have a questionable reliability. However, the results for these two outcomes align with the results for the other four outcomes, which have a good reliability.

We analyzed one specific cohort of children in one country. Some studies indicate that the country context could affect the impact of parenting on child development (e.g. Bradbury et al., 2015). For instance, more extensive public childcare in Germany compared to the United States could reduce the influence of parenting and especially, socio-economic differences in such influences, as children may receive support lacking in the family in the public institutions. Further research should more intensively study cross-country variation in the effects of parenting on child development.

In general, however, our findings, together with the evidence from previous studies, support the view that the effects of parenting on children may be heterogeneous. First, there may be an age gradient in the effects of parenting. While previous studies mostly found that parenting had effects on child development in early childhood, we found no effects of parenting styles, parental activities, and extracurricular activities in early adolescence on noncognitive skills. This possible negative age gradient in the effects of parenting on the skill development of children has implications for the timing of policy interventions. In line with Cunha and Heckman (2008), our study indicates that such interventions are more promising in early childhood than in adolescence. Second, there may be differences across different types of skills. We focused on noncognitive skills as the outcome but could not take into account the effects parenting on cognitive skills in adolescence.

## Supplemental Material

sj-pdf-1-asj-10.1177_00016993211051958 - Supplemental material for The effects of parenting on early adolescents’ noncognitive skills: Evidence from a sample of twins in Germany Click here for additional data file. Supplemental material, sj-pdf-1-asj-10.1177_00016993211051958 for The effects of parenting on early adolescents’ noncognitive skills: Evidence from a sample of twins in Germany by Michael Grätz, Volker Lang and Martin Diewald in Acta Sociologica

## Notes

1. This behavior could vary by family socioeconomic background. One study found socioeconomically advantaged families to reinforce ability differences among twins (Grätz and Torche, 2016) but two other studies found socioeconomically advantaged families to compensate for differences in endowments among siblings (Hsin, 2012; Restrepo, 2016).

2. The shares of missing information for all measures are reported in Table S1 in the *Online Supplement*. Most information is missing on three of our outcome variables: self-efficacy, self-esteem, and locus of control. For the explanatory and the control variables the shares of missing information are mostly low. Differentiations of the shares of missing information by parental education and occupation (columns 3 to 6 of Table S1 in the *Online Supplement*) show that information tends to be more often missing for children with parents with lower levels of education and occupation. These differences are slightly larger for the outcomes self-efficacy and self-esteem collected in the second survey wave. However, overall the differences in shares of missing information by family socioeconomic background are rather small.

3. The correlations between children's and parents’ reports for each parenting style are shown in Table S5 in the *Online Supplement*. We find stronger correlations between children's reports on mother's and father's parenting styles than between parent's self-reports on their parenting styles, indicating that children perceive the parenting styles less differentiated than their parents. However, this overall conclusion does not hold for the within-twin-pair differences in parenting styles. Here, parents report less differences than their children (see Table 2). Additionally, we find only moderate correlations between children's and parents’ self-reports on parenting styles for both mothers and fathers. Table S4 in the *Online Supplement* reports also the reliabilities of the scales using only parental or children's reports on parenting styles. These are lower than those using the combined measure of all four parenting style reports. We therefore use only the latter in our analysis, which is also in line with suggestions in previous research (e.g., Bögels and van Melick, 2004).

4. In addition, we report the within-twin pair correlations for all measures included in our analysis in the last column of Table 1. For those measures varying within twin pairs, we find the lowest correlations for self-efficacy and the highest for extracurricular activities. Most of the correlations are lower for DZ compared to MZ twin pairs indicating genetic influences on these measure. Given these results it is advisable to analyse DZ and MZ twins separately. The MZ twin fixed effects models control for the influences of genetic differences.

5. Furthermore, we assessed whether the means of parenting differed between those parents who treat their twin children differently and those who do not, and if there are differences in the shares of parents treating their twin children similar or different by parental education or occupation. These additional descriptive results are reported in Tables S8 and S9 in the *Online Supplement*. The results show no statistically significant correlations between the means of parenting and the differences in parenting within twin pairs (Table S8). Moreover, we did not find substantial differences in the shares of parents treating their children similar or different by family socioeconomic background (Table S9). Most of the differences in shares are smaller than 0.10. The largest difference is found in parental activities with respect to parental education for MZ twin pairs. Here, the share of parents with high education treating their twin children differently is 0.15 larger than the respective share treating them similar. Taken together, the results indicate no systematic relations between means and differences in parenting and family background. These findings help to counteract worries about the generalizability of our study, which is based on twin fixed-effects analyses focusing on differences within twin pairs.

6. The following power analysis demonstrates that our sample is large enough to find effects of parenting on noncognitive skills. With alpha set at 0.05 and a power equal to 0.80, our data allows us to depict an effect size equal to 0.005 in the models using DZ twins (*N* = 1,532 twins) and equal to 0.007 in the models using MZ twins (*N* = 1,080 twins) when we use our largest sample for academic self-concept (Faul et al., 2009). Our sample is smallest for self-efficacy. With alpha set at 0.05 and a power equal to 0.80, our data allows us to depict an effect size equal to 0.007 in the models using DZ twins (*N* = 1,148 twins) and equal to 0.010 in the models using MZ twins (*N* = 760 twins).
