# Supplementary material for: The effects of parenting on early adolescents’ noncognitive skills: Evidence from a sample of twins in Germany
Source: Acta Sociol. 2021 Nov 15;65(4):398–419. doi: 10.1177/00016993211051958 (PMC9535998; doi:10.1177/00016993211051958)
Supplement: sj-pdf-1-asj-10.1177_00016993211051958 - Supplemental material for The effects of parenting on early adolescents’ noncognitive skills: Evidence from a sample of twins in Germany [file sj-pdf-1-asj-10.1177_00016993211051958.pdf]

ONLINE SUPPLEMENT TO

Grätz, Michael, Volker Lang, and Martin Diewald. 2021. The Effects of Parenting on Early Adolescents' Noncognitive Skills: Evidence from a Sample of Twins in Germany. *Acta Sociologica,* forthcoming.

This version: June 22, 2021

Table S1. Shares of Missing Information Overall and by Parental Education and Occupation.

	Overall	Low Parental Education	High Parental Education	Low Parental Occupation	High Parental Occupation
Panel A: Dizygotic twins					
Academic self-concept	0.03	0.04	0.02	0.03	0.02
Intrinsic motivation	0.03	0.04	0.02	0.03	0.03
Learning motivation	0.04	0.06	0.02	0.03	0.04
Self-efficacy	0.27	0.33	0.23	0.34	0.23
Self-esteem	0.16	0.18	0.15	0.20	0.14
Locus of control	0.17	0.16	0.17	0.16	0.17
Parental warmth	0	0	0	0	0
Parental control	0	0	0	0	0
Parental activities	0.09	0.10	0.09	0.05	0.11
Extracurricular activities	0	0	0	0	0
IQ	0.06	0.07	0.05	0.05	0.06
Birth weight (in kg)	0.08	0.09	0.08	0.09	0.08
Academic self-concept, wave 1	0.04	0.04	0.04	0.04	0.04
Intrinsic motivation, wave 1	0.02	0.03	0.01	0.02	0.02
Learning motivation, wave 1	0.08	0.11	0.06	0.09	0.07
Self-efficacy, wave 1	0.02	0.01	0.02	0.01	0.02
Panel B: Monozygotic twins					
Academic self-concept	0.01	0.02	0.00	0.02	0.00
Intrinsic motivation	0.01	0.02	0.00	0.02	0.00
Learning motivation	0.02	0.03	0.01	0.04	0.01
Self-efficacy	0.30	0.32	0.28	0.30	0.30
Self-esteem	0.15	0.15	0.15	0.15	0.15
Locus of control	0.19	0.20	0.18	0.19	0.19
Parental warmth	0	0	0	0	0
Parental control	0	0	0	0	0
Parental activities	0.11	0.11	0.11	0.13	0.10
Extracurricular activities	0	0	0	0	0
IQ	0.07	0.07	0.06	0.08	0.05
Birth weight (in kg)	0.09	0.05	0.13	0.08	0.10
Academic self-concept, wave 1	0.02	0.02	0.02	0.03	0.02
Intrinsic motivation, wave 1	0.03	0.03	0.02	0.02	0.03
Learning motivation, wave 1	0.09	0.09	0.09	0.10	0.08
Self-efficacy, wave 1	0.02	0.02	0.02	0.03	0.01

Source: TwinLife, version 4.0.0 (doi:10.4232/1.13539).

Table S2. Twin Fixed-Effects Models of the Effects of Parenting on Children's Noncognitive Skills, Controlling for IQ, Birth Weight, and Noncognitive Skills at Wave 1, OLS Regressions with Listwise Deletion of Observations with Missing Values.

	(1) Academic Self-Concept	(2) Intrinsic Motivation	(3) Learning Motivation	(4) Self-Efficacy	(5) Self-Esteem	(6) Locus of Control
	Panel A: Dizygotic twins					
Parental warmth	–0.07	–0.03	0.06	0.08	0.24†	0.20
	(0.12)	(0.11)	(0.12)	(0.14)	(0.12)	(0.13)
Parental control	0.03	0.04	–0.04	0.10	–0.12	–0.04
	(0.10)	(0.09)	(0.14)	(0.10)	(0.11)	(0.10)
Parental warmth	–0.12	–0.02	0.04	0.05	0.09	–0.07
X Parental control	(0.08)	(0.08)	(0.10)	(0.08)	(0.08)	(0.08)
Parental activities	–0.01	–0.07	0.01	–0.09	–0.06	0.01
	(0.08)	(0.09)	(0.10)	(0.09)	(0.09)	(0.07)
Extracurricular	0.20	0.08	0.14	0.23†	0.06	0.33*
activities	(0.12)	(0.16)	(0.17)	(0.12)	(0.15)	(0.14)
N (twins)	368	368	368	368	368	368
	Panel B: Monozygotic twins					
Parental warmth	0.25*	0.13	–0.03	0.27†	–0.02	0.00
	(0.12)	(0.10)	(0.12)	(0.14)	(0.14)	(0.11)
Parental control	–0.28†	0.08	–0.01	0.05	–0.04	–0.03
	(0.14)	(0.13)	(0.14)	(0.17)	(0.13)	(0.12)
Parental warmth	0.17†	–0.08	0.06	0.13	–0.04	0.04
X Parental control	(0.09)	(0.08)	(0.10)	(0.11)	(0.11)	(0.09)
Parental activities	–0.07	–0.02	–0.03	–0.06	–0.03	0.24*
	(0.11)	(0.08)	(0.10)	(0.10)	(0.11)	(0.11)
Extracurricular	0.12	–0.03	–0.03	0.51†	0.37	0.06
activities	(0.24)	(0.19)	(0.26)	(0.28)	(0.28)	(0.18)
N (twins)	232	232	232	232	232	232

Notes: All variables are z-standardized. Cluster-robust standard errors in parentheses. All models control for IQ, birth weight, academic self-concept at wave 1, intrinsic motivation at wave 1, learning motivation at wave 1, and self-efficacy at wave 1 (controls not shown).
Source: TwinLife, version 4.0.0 (doi:10.4232/1.13539).
† p < 0.10; * p < 0.05; ** p < 0.01

Table S3. Questions and Response Scales Used to Measure Noncognitive Skills.

Construct Items	Question Text in English	Question Text in German	Response Scale
Academic self-concept			
spa0200	1) I am ... in school.	1) Ich bin für die Schule ...	1: not talented<br>2, 3, 4<br>5: very talented
spa0201	2) I know … in school.	2) Ich kann in der Schule …	1: just a little<br>2, 3, 4<br>5: a lot
spa0202	3) In school, many assignments are ... for me.	3) In der Schule fallen mir viele Aufgaben …	1: easy<br>2, 3, 4<br>5: difficult
Intrinsic motivation			1: doesn't apply at all<br>2: doesn't apply
imo0200	1) I like doing the things that I learn at school.	1) Ich mache die Dinge, die ich in der Schule lerne, gerne.	3: nor<br>4: applies<br>5: applies completely
imo0201	2) School is fun.	2) Schule macht mir Spaß.	
imo0202	3) I find the things that I learn at school interesting.	3) Was ich in der Schule lerne, finde ich interessant.	
Learning motivation	At school, I am interested in …	In der Schule geht es mir darum …	1: doesn't apply at all<br>2: doesn't apply
imo0500	1) learning something interesting.	1) etwas Interessantes zu lernen.	3: nor<br>4: applies
imo0501	2) understand difficult things / getting motivated to think about things.	2) schwierige Dinge verstehen / zum Nachdenken angeregt zu werden.	5: applies completely
imo0502	3) learn as much as possible / gaining a thorough understanding of content / really understanding something.	3) so viel wie möglich lernen / ein tieferes Verständnis für die Inhalte zu erwerben / etwas wirklich zu verstehen.	
Self-efficacy	To what extent do you agree with these statements?	Wie sehr stimmst Du folgenden Aussagen zu?	1: strongly disagree<br>2: disagree
sef0100	1) I can rely on my own abilities in difficult situations.	1) In schwierigen Situationen kann ich mich auf meine Fähigkeiten verlassen.	3: neutral<br>4: agree<br>5: strongly agree
sef0101	2) I am able to solve most problems on my own.	2) Die meisten Probleme kann ich aus eigener Kraft gut meistern.	
sef0102	3) I can usually solve even challenging and complex tasks well.	3) Auch anstrengende und komplizierte Aufgaben kann ich in der Regel gut lösen.	
Self-esteem	To what extent do you agree with these statements?	Wie sehr stimmst Du folgenden Aussagen zu?	1: strongly disagree<br>2: disagree
ses0100	1) At times I think that I am no good at all.	1) Manchmal denke ich, dass ich wertlos bin.	3: neutral<br>4: agree
ses0101	2) I take a positive attitude toward myself.	2) Ich mag mich so, wie ich bin.	5: strongly agree
ses0102	3) On the whole, I am satisfied with myself.	3) Alles in allem bin ich mit mir selbst zufrieden.	

Locus of control	To what extent do you think each statement applies to you personally.	Bitte geben Sie bei jeder Aussage an, inwieweit diese auf Sie persönlich zutrifft.	1: doesn’t apply at all<br>2: doesn’t apply<br>3: nor<br>4: applies<br>5: applies completely
loc0100	1) I'm my own boss.	1) Ich habe mein Leben selbst in der Hand.	
loc0101	2) If I work hard, I will succeed.	2) Wenn ich mich anstrenge, werde ich auch Erfolg haben.	
loc0102 (coding reversed)	3) Whether at work or in my private life: What I do is mainly determined by others.	3) Egal ob privat oder im Beruf: Mein Leben wird zum großen Teil von anderen bestimmt.	
loc0103 (coding reversed)	4) Fate often gets in the way of my plans.	4) Meine Pläne werden oft vom Schicksal durchkreuzt.	

Source: TwinLife, version 4.0.0 (doi:10.4232/1.13539).

Table S4. Scale Reliabilities for Noncognitive Skills and Different Reports of Parenting Styles.

	Number of items	Cronbach's α
NONCOGNITIVE SKILLS		
Academic-self concept	3	0.67
Intrinsic motivation	3	0.80
Learning motivation	3	0.74
Self-efficacy	3	0.73
Self-esteem	3	0.75
Locus of control	4	0.64
PARENTING STYLES		
Warmth of mother, mother's report	4	0.77
Warmth of father, father's report	4	0.83
Warmth of parents, parent's reports	8	0.76
Warmth of mother, children's report	4	0.78
Warmth of father, children's report	4	0.86
Warmth of parents, children's reports	8	0.87
Warmth of parents, parent's and children's reports	16	0.85
Control of mother, mother's report	3	0.64
Control of father, father's report	3	0.63
Control of parents, parent's reports	6	0.68
Control of mother, children's report	3	0.53
Control of father, children's report	3	0.62
Control of parents, children's reports	6	0.76
Control of parents, parent's and children's reports	12	0.76

Source: TwinLife, version 4.0.0 (doi:10.4232/1.13539).

Table S5. Correlations between the Different Reports of Parenting Styles.

	1)	2)	3)	4)
PARENTAL WARMTH				
Panel A: Dizygotic twins				
1) Warmth of mother, mother's report	1			
2) Warmth of father, father's report	0.18**	1		
3) Warmth of mother, children's report	0.33**	0.15**	1	
4) Warmth of father, children's report	0.18**	0.24**	0.65**	1
Panel B: Monozygotic twins				
1) Warmth of mother, mother's report	1			
2) Warmth of father, father's report	0.13**	1		
3) Warmth of mother, children's report	0.22**	0.06	1	
4) Warmth of father, children's report	0.08	0.28**	0.55**	1
PARENTAL CONTROL				
Panel A: Dizygotic twins				
1) Control of mother, mother's report	1			
2) Control of father, father's report	0.26**	1		
3) Control of mother, children's report	0.25**	0.17**	1	
4) Control of father, children's report	0.17**	0.23**	0.64**	1
Panel B: Monozygotic twins				
1) Control of mother, mother's report	1			
2) Control of father, father's report	0.34**	1		
3) Control of mother, children's report	0.34**	0.22**	1	
4) Control of father, children's report	0.23**	0.22**	0.72**	1

Source: TwinLife, version 4.0.0 (doi:10.4232/1.13539).

† $p < 0.10$; * $p < 0.05$; ** $p < 0.01$

Table S6. Questions and Response Scales Used to Measure Parenting.

Construct Items	Question Text in English	Question Text in German	Response Scale
Parental warmth (parent's report)[a]	How often do the following things typically happen between you and [name of child]?	Wie häufig kommen folgende Dinge typischerweise zwischen Ihnen und [Name des Kindes] vor?	1: not at all<br>2: about once a month<br>3: about once a week<br>4: several times a week<br>5: (almost) daily
par0100	1) You show [name of child] with words and gestures that you like him/her.	1) Sie zeigen [Name des Kindes] mit Worten und Gesten, dass Sie ihn/sie gerne haben.	
par0101	2) You praise [name of child].	2) Sie loben [Name des Kindes].	
par0102	3) You cheer up [name of child] when he/she is sad.	3) Sie trösten [Name des Kindes].	
par0103	4) You give [name of child] advice regarding his/her personal problems.	4) Sie unterstützen [Name des Kindes], wenn er/sie ein Problem hat.	
Parental control (parent's report)[a]	How often do the following things typically happen between you and [name of child]?	Wie häufig kommen folgende Dinge typischerweise zwischen Ihnen und [Name des Kindes] vor?	1: not at all<br>2: about once a month<br>3: about once a week<br>4: several times a week<br>5: (almost) daily
par0104	1) If [name of child] does something against your will, you punish him/her.	1) Wenn [Name des Kindes] etwas gegen Ihren Willen tut, bestrafen Sie ihn/sie.	
par0105	2) You are disappointed or sad if [name of child] behaved badly.	2) Sie sind enttäuscht und traurig, wenn sich [Name des Kindes] schlecht benommen hat.	
par0106	3) You make it clear to [name of child] that he/she is not to break the rules or question your decisions.	3) Sie geben [Name des Kindes] zu verstehen, dass er/sie sich Ihren Anordnungen und Entscheidungen nicht widersetzen soll.	
Parental activities (children's report)	How often have your parents or other members of your family taken part in the following activities with you during the last four weeks?	Wie oft haben Deine Eltern oder jemand anderes aus der Familie in den letzten 4 Wochen gemeinsam mit Dir folgende Aktivitäten unternommen?	1: not at all (coded as "0")<br>2: about once a month (coded as "1")<br>3: about once a week (coded as "4")<br>4: several times a week (coded as "8")<br>5: (almost) daily (coded as "8")
acc0100	1) Singing and making music.	1) Singen oder musizieren.	
acc0101	2) Reading books and talking about books.	2) Bücher lesen oder sich über Bücher unterhalten.	
acc0102	3) Sports.	3) Sportliche Aktivitäten.	
acc0103	4) Walks, excursions, visiting other families.	4) Spaziergänge, Ausflüge, Besuche von anderen Familien.	
acc0104	5) Visit a theatre, museum, exhibition, classical concert or similar.	5) Besuch eines Theaters, Museums, einer Ausstellung, eines klassisches Konzerts oder Ähnliches.	

Extra-curricular activities (parent's report)	Below is a list of groups that one might be active in. For each one, please state how often [parent report: "name of twin X" takes part; twin report: you take part].	Im Folgenden sehen Sie eine Reihe von Gruppen, in denen man aktiv sein kann. Bitte geben Sie an, in welchem Ausmaß [parent report: „Name von Zwilling X"; twin report: Sie] in den jeweiligen Gruppen aktiv [parent report: ist; twin report: sind].	1: every week (coded as "4")<br>2: every month (coded as "1")<br>3: less than once a month (coded as "0")<br>4: never (coded as "0")
sop0100	1) Sports club.	1) Sportverein.	
sop0101	2) Choir, music society, theater group.	2) Gesangsverein, Musikverein, Theatergruppe.	
sop0102	3) Church group, religious group.	3) Kirchliche Gruppe, religiöse Gruppe.	
sop0103	4) Trade union, occupational association, student council.	4) Gewerkschaft, Berufsverband, Schülervertretung.	
sop0104	5) Volunteer fire department, technical relief, lifeguards.	5) Freiwillige Feuerwehr, THW, DLRG.	
sop0105	6) Local organizations, marksmen club.	6) Heimatverein, Bürgerverein, Schützenverein.	
sop1050	7) Other association.	7) Anderer Verein.	

[a] Questions, items, and response scales for children's reports are analogous to parent's reports.
Source: TwinLife, version 4.0.0 (doi:10.4232/1.13539).

Table S7. Correlations between the Different Measures of Parenting.

	Parental Warmth	Parental Control	Parental Activities	Extracurricular Activities
	Panel A: Dizygotic twins			
Parental warmth	1			
Parental control	–0.12**	1		
Parental activities	0.20**	–0.08*	1	
Extracurricular activities	0.01	–0.02	0.16**	1
	Panel B: Monozygotic twins			
Parental warmth	1			
Parental control	–0.13**	1		
Parental activities	0.16**	–0.06	1	
Extracurricular activities	0.07	–0.07	0.28**	1

Source: TwinLife, version 4.0.0 (doi:10.4232/1.13539).
† p < 0.10; * p < 0.05; ** p < 0.01

Table S8. Descriptive Statistics of Parenting by Differences in Parenting within Twin Pairs.

	No Differences:			Differences:			Correlation of Mean and Difference
	N_{pairs}	Mean	SD	N_{pairs}	Mean	SD	
Panel A: Dizygotic twins							
Parental warmth	39	4.61	0.33	354	4.36	0.36	–0.03
Parental control	24	2.60	0.38	369	2.78	0.44	–0.06
Parental activities	36	11.97	9.33	320	13.78	7.39	0.00
Extracurricular activities	293	5.25	3.23	100	5.69	2.73	0.04
Panel B: Monozygotic twins							
	N_{pairs}	Mean	SD	N_{pairs}	Mean	SD	Correlation of Mean and Difference
Parental warmth	36	4.58	0.33	236	4.35	0.36	–0.02
Parental control	21	2.76	0.50	251	2.60	0.64	–0.01
Parental activities	33	10.70	9.11	209	13.13	7.54	0.07
Extracurricular activities	236	5.17	3.44	36	6.46	3.48	0.02

Note: The last column reports the correlations between the mean per twin pair and the difference within the twin pairs. We tested the statistical significance of these correlations; however, in no case the correlation was statistically significant different from zero.

Source: TwinLife, version 4.0.0 (doi:10.4232/1.13539).

Table S9. Share of Respondents with High Parental Education and Occupation by Differences in Parenting within Twin Pairs.

	No Differences: Share High Parental Education	Share High Parental Occupation	Differences: Share High Parental Education	Share High Parental Occupation
Panel A: Dizygotic twins				
Parental warmth	0.67	0.74	0.59	0.66
Parental control	0.58	0.51	0.60	0.68
Parental activities	0.61	0.61	0.59	0.68
Extracurricular activities	0.60	0.67	0.59	0.68
Panel B: Monozygotic twins				
Parental warmth	0.61	0.61	0.50	0.59
Parental control	0.44	0.53	0.53	0.60
Parental activities	0.39	0.52	0.54	0.61
Extracurricular activities	0.52	0.59	0.53	0.61

Source: TwinLife, version 4.0.0 (doi:10.4232/1.13539).

Table S10. Twin Fixed-Effects Models of the Effects of Parenting on Children's Academic Self-Concept, Controlling for IQ, Birth Weight, and Noncognitive Skills at Wave 1.

	(1)	(2)	(3)	(4)	(5)
		Panel A: Dizygotic twins			
Parental warmth	0.05		0.05		
	(0.07)		(0.07)		
Parental control		–0.01	0.00		
		(0.06)	(0.06)		
Parental warmth			–0.08		
X Parental control			(0.05)		
Parental activities				0.06	
				(0.06)	
Extracurricular activities					0.04
					(0.09)
N (twins)	766	766	766	766	766
		Panel B: Monozygotic twins			
Parental warmth	0.13		0.11		
	(0.08)		(0.08)		
Parental control		–0.13	–0.15†		
		(0.08)	(0.08)		
Parental warmth			0.07		
X Parental control			(0.07)		
Parental activities				–0.12†	
				(0.07)	
Extracurricular activities					0.00
					(0.12)
N (twins)	540	540	540	540	540

Notes: All variables are z-standardized. Cluster-robust standard errors in parentheses. All models control for IQ, birth weight, academic self-concept at wave 1, intrinsic motivation at wave 1, learning motivation at wave 1, and self-efficacy at wave 1 (controls not shown).
Source: TwinLife, version 4.0.0 (doi:10.4232/1.13539).

Table S11. Twin Fixed-Effects Models of the Effects of Parenting on Children's Intrinsic Motivation, Controlling for IQ, Birth Weight, and Noncognitive Skills at Wave 1.

	(1)	(2)	(3)	(4)	(5)
			Panel A: Dizygotic twins		
Parental warmth	–0.05		–0.05		
	(0.07)		(0.07)		
Parental control		0.00	0.00		
		(0.06)	(0.06)		
Parental warmth			0.03		
X Parental control			(0.05)		
Parental activities				–0.01	
				(0.06)	
Extracurricular activities					0.01
					(0.09)
N (twins)	764	764	764	764	764
			Panel B: Monozygotic twins		
Parental warmth	0.07		0.10		
	(0.07)		(0.06)		
Parental control		–0.05	–0.04		
		(0.09)	(0.08)		
Parental warmth			–0.10		
X Parental control			(0.07)		
Parental activities				–0.07	
				(0.07)	
Extracurricular activities					–0.22
					(0.19)
N (twins)	540	540	540	540	540

Notes: All variables are z-standardized. Cluster-robust standard errors in parentheses. All models control for IQ, birth weight, academic self-concept at wave 1, intrinsic motivation at wave 1, learning motivation at wave 1, and self-efficacy at wave 1 (controls not shown).
Source: TwinLife, version 4.0.0 (doi:10.4232/1.13539).
† $p < 0.10$; * $p < 0.05$; ** $p < 0.01$

Table S12. Twin Fixed-Effects Models of the Effects of Parenting on Children's Learning Motivation, Controlling for IQ, Birth Weight, and Noncognitive Skills at Wave 1.

	(1)	(2)	(3)	(4)	(5)
			Panel A: Dizygotic Twins		
Parental warmth	0.04		0.05		
	(0.07)		(0.07)		
Parental control		0.02	0.00		
		(0.08)	(0.08)		
Parental warmth			0.06		
X Parental control			(0.06)		
Parental activities				0.04	
				(0.07)	
Extracurricular activities					–0.06
					(0.10)
N (twins)	758	758	758	758	758
			Panel B: Monozygotic twins		
Parental warmth	0.02		0.01		
	(0.07)		(0.07)		
Parental control		–0.02	–0.03		
		(0.07)	(0.07)		
Parental warmth			0.07		
X Parental control			(0.06)		
Parental activities				–0.09	
				(0.07)	
Extracurricular activities					–0.03
					(0.10)
N (twins)	534	534	534	534	534

Notes: All variables are z-standardized. Cluster-robust standard errors in parentheses. All models control for IQ, birth weight, academic self-concept at wave 1, intrinsic motivation at wave 1, learning motivation at wave 1, and self-efficacy at wave 1 (controls not shown).
Source: TwinLife, version 4.0.0 (doi:10.4232/1.13539).
† $p < 0.10$; * $p < 0.05$; ** $p < 0.01$

Table S13. Twin Fixed-Effects Models of the Effects of Parenting on Children's Self-Efficacy, Controlling for IQ, Birth Weight, and Noncognitive Skills at Wave 1.

	(1)	(2)	(3)	(4)	(5)
			Panel A: Dizygotic twins		
Parental warmth	0.11		0.10		
	(0.09)		(0.09)		
Parental control		0.12	0.11		
		(0.08)	(0.08)		
Parental warmth X Parental control			0.04		
			(0.07)		
Parental activities				–0.03	
				(0.08)	
Extracurricular activities					0.10
					(0.11)
N (twins)	574	574	574	574	574
			Panel B: Monozygotic twins		
Parental warmth	0.20*		0.16		
	(0.10)		(0.10)		
Parental control		0.15	0.12		
		(0.11)	(0.11)		
Parental warmth X Parental control			0.12		
			(0.08)		
Parental activities				–0.11	
				(0.08)	
Extracurricular activities					0.25
					(0.19)
N (twins)	380	380	380	380	380

Notes: All variables are z-standardized. Cluster-robust standard errors in parentheses. All models control for IQ, birth weight, academic self-concept at wave 1, intrinsic motivation at wave 1, learning motivation at wave 1, and self-efficacy at wave 1 (controls not shown).
Source: TwinLife, version 4.0.0 (doi:10.4232/1.13539).
† $p < 0.10$; * $p < 0.05$; ** $p < 0.01$

Table S14. Twin Fixed-Effects Models of the Effects of Parenting on Children's Self-Esteem, Controlling for IQ, Birth Weight, and Noncognitive Skills at Wave 1.

	(1)	(2)	(3)	(4)	(5)
			Panel A: Dizygotic twins		
Parental warmth	0.14*		0.15*		
	(0.07)		(0.07)		
Parental control		–0.09	–0.11		
		(0.08)	(0.08)		
Parental warmth			0.08		
X Parental control			(0.06)		
Parental activities				–0.06	
				(0.07)	
Extracurricular activities					–0.02
					(0.11)
N (twins)	660	660	660	660	660
			Panel B: Monozygotic twins		
Parental warmth	–0.08		–0.09		
	(0.10)		(0.10)		
Parental control		0.00	0.01		
		(0.09)	(0.09)		
Parental warmth			0.02		
X Parental control			(0.09)		
Parental activities				0.04	
				(0.08)	
Extracurricular activities					–0.05
					(0.14)
N (twins)	462	462	462	462	462

Notes: All variables are z-standardized. Cluster-robust standard errors in parentheses. All models control for IQ, birth weight, academic self-concept at wave 1, intrinsic motivation at wave 1, learning motivation at wave 1, and self-efficacy at wave 1 (controls not shown).
Source: TwinLife, version 4.0.0 (doi:10.4232/1.13539).
† $p < 0.10$; * $p < 0.05$; ** $p < 0.01$

Table S15. Twin Fixed-Effects Models of the Effects of Parenting on Children's Locus of Control, Controlling for IQ, Birth Weight, and Noncognitive Skills at Wave 1.

	(1)	(2)	(3)	(4)	(5)
			Panel A: Dizygotic twins		
Parental warmth	0.11		0.11		
	(0.09)		(0.09)		
Parental control		–0.08	–0.09		
		(0.07)	(0.07)		
Parental warmth			–0.04		
X Parental control			(0.06)		
Parental activities				0.09	
				(0.06)	
Extracurricular activities					0.08
					(0.11)
N (twins)	656	656	656	656	656
			Panel B: Monozygotic twins		
Parental warmth	–0.07		–0.08		
	(0.11)		(0.11)		
Parental control		0.07	0.07		
		(0.10)	(0.10)		
Parental warmth			0.04		
X Parental control			(0.07)		
Parental activities				0.04	
				(0.09)	
Extracurricular activities					0.11
					(0.14)
N (twins)	440	440	440	440	440

Notes: All variables are z-standardized. Cluster-robust standard errors in parentheses. All models control for IQ, birth weight, academic self-concept at wave 1, intrinsic motivation at wave 1, learning motivation at wave 1, and self-efficacy at wave 1 (controls not shown).
Source: TwinLife, version 4.0.0 (doi:10.4232/1.13539).† p < 0.10; * p < 0.05; ** p < 0.01

Table S16. Twin Fixed-Effects Models of the Effects of Parenting on Children's Noncognitive Skills, Ensembles of Parenting.

	(1) Academic Self-Concept	(2) Intrinsic Motivation	(3) Learning Motivation	(4) Self-Efficacy	(5) Self-Esteem	(6) Locus of Control
Panel A: Dizygotic twins						
Parental warmth	0.10	0.07	0.16*	0.15	0.19**	0.15†
	(0.07)	(0.07)	(0.07)	(0.09)	(0.06)	(0.08)
Parental control	–0.07	–0.02	0.00	0.03	–0.13†	–0.11
	(0.07)	(0.07)	(0.08)	(0.09)	(0.08)	(0.07)
Parental warmth	–0.06	0.06	0.08	0.04	0.07	–0.03
X Parental control	(0.06)	(0.05)	(0.06)	(0.07)	(0.05)	(0.06)
Parental activities	0.10	0.00	0.05	0.04	–0.04	0.10
	(0.07)	(0.06)	(0.07)	(0.09)	(0.07)	(0.06)
Parental warmth X	0.00	–0.02	–0.02	–0.01	0.00	0.00
Parental activities	(0.03)	(0.01)	(0.02)	(0.03)	(0.02)	(0.02)
Parental control X	–0.01	–0.03†	–0.01	–0.03	0.04	–0.03
Parental activities	(0.03)	(0.02)	(0.02)	(0.03)	(0.02)	(0.02)
Extracurricular	0.03	0.02	–0.06	0.05	–0.03	0.09
activities	(0.09)	(0.10)	(0.11)	(0.12)	(0.11)	(0.10)
Parental warmth X	0.00	0.02	0.01	0.03	0.02	0.01
Extracurricular activities	(0.02)	(0.02)	(0.02)	(0.03)	(0.01)	(0.02)
Parental control X	0.01	0.02	0.04†	0.01	0.00	0.00
Extracurricular activities	(0.02)	(0.02)	(0.02)	(0.03)	(0.02)	(0.02)
N (twins)	766	764	758	574	660	656
Panel B: Monozygotic twins						
Parental warmth	0.14†	0.13†	0.06	0.21*	–0.07	–0.03
	(0.08)	(0.07)	(0.07)	(0.09)	(0.10)	(0.10)
Parental control	–0.16†	–0.08	–0.06	0.12	–0.01	0.01
	(0.08)	(0.08)	(0.07)	(0.11)	(0.08)	(0.10)
Parental warmth	0.08	–0.09	0.06	0.07	0.01	0.03
X Parental control	(0.07)	(0.06)	(0.06)	(0.08)	(0.08)	(0.08)
Parental activities	–0.07	–0.01	–0.04	–0.03	0.05	0.08
	(0.07)	(0.06)	(0.07)	(0.07)	(0.08)	(0.09)
Parental warmth X	0.04†	–0.02	0.01	0.00	–0.01	–0.02
Parental activities	(0.02)	(0.02)	(0.02)	(0.02)	(0.02)	(0.03)
Parental control X	0.02	0.00	0.01	–0.02	–0.03	–0.01
Parental activities	(0.02)	(0.02)	(0.02)	(0.02)	(0.02)	(0.03)
Extracurricular	0.01	–0.20	–0.01	0.19	–0.02	0.19
activities	(0.12)	(0.19)	(0.10)	(0.20)	(0.13)	(0.14)
Parental warmth X	–0.01	0.00	0.01	–0.02	0.02	0.03
Extracurricular activities	(0.02)	(0.02)	(0.01)	(0.02)	(0.02)	(0.02)
Parental control X	0.00	0.03†	0.01	–0.01	0.01	0.01
Extracurricular activities	(0.02)	(0.01)	(0.01)	(0.02)	(0.02)	(0.02)
N (twins)	540	540	534	380	462	440

Notes: All variables are z-standardized. Cluster-robust standard errors in parentheses.
Source: TwinLife, version 4.0.0 (doi:10.4232/1.13539).
† p < 0.10; * p < 0.05; ** p < 0.01

Table S17. Twin Fixed-Effects Models of the Effects of Parenting on Children's Noncognitive Skills, Interactions with Parental Occupation, Controlling for IQ, Birth Weight, and Noncognitive Skills at Wave 1.

	(1) Academic Self-Concept	(2) Intrinsic Motivation	(3) Learning Motivation	(4) Self-Efficacy	(5) Self-Esteem	(6) Locus of Control
Panel A: Dizygotic twins						
Parental warmth	0.23*	–0.05	0.14	0.20	0.27*	0.03
	(0.10)	(0.10)	(0.14)	(0.17)	(0.12)	(0.11)
Parental control	–0.05	0.01	–0.15	0.04	–0.24*	–0.13
	(0.10)	(0.10)	(0.15)	(0.13)	(0.12)	(0.11)
Parental warmth X Parental control	–0.07	0.03	0.07	0.05	0.08	–0.04
	(0.05)	(0.05)	(0.06)	(0.06)	(0.06)	(0.06)
Parental activities	0.02	–0.03	–0.01	0.01	–0.04	0.08
	(0.10)	(0.09)	(0.12)	(0.13)	(0.07)	(0.12)
Extracurricular activities	0.19	0.10	–0.20	0.21	0.11	–0.04
	(0.14)	(0.17)	(0.22)	(0.18)	(0.14)	(0.23)
Parental warmth X High parental occupation	–0.26*	0.01	–0.15	–0.14	–0.16	0.14
	(0.13)	(0.12)	(0.16)	(0.19)	(0.15)	(0.15)
Parental control X High parental occupation	0.06	–0.02	0.25	0.11	0.22	0.06
	(0.12)	(0.12)	(0.17)	(0.17)	(0.15)	(0.15)
Parental warmth X Parental control X High parental occupation	–0.08	–0.01	0.08	–0.06	–0.10†	–0.12†
	(0.05)	(0.05)	(0.05)	(0.07)	(0.06)	(0.06)
Parental activities X High parental occupation	0.05	0.03	0.06	–0.07	–0.03	0.01
	(0.12)	(0.12)	(0.14)	(0.16)	(0.13)	(0.14)
Extracurricular activities X High parental occupation	–0.19	–0.13	0.19	–0.15	–0.18	0.18
	(0.17)	(0.20)	(0.25)	(0.23)	(0.20)	(0.26)
N (twins)	766	764	758	574	660	656
Panel B: Monozygotic twins						
Parental warmth	0.16	0.14	0.10	0.36†	–0.09	–0.14
	(0.12)	(0.11)	(0.10)	(0.19)	(0.18)	(0.19)
Parental control	–0.22†	–0.06	–0.01	0.15	0.06	0.14
	(0.13)	(0.13)	(0.09)	(0.19)	(0.14)	(0.20)
Parental warmth X Parental control	0.08	–0.10	0.09	0.15†	0.03	0.05
	(0.06)	(0.07)	(0.06)	(0.08)	(0.08)	(0.08)
Parental activities	–0.16	–0.04	–0.25*	–0.15	0.04	–0.06
	(0.13)	(0.12)	(0.12)	(0.14)	(0.12)	(0.17)
Extracurricular activities	0.05	–0.60*	–0.09	0.48	–0.13	0.19
	(0.19)	(0.29)	(0.19)	(0.35)	(0.18)	(0.35)
Parental warmth X High parental occupation	–0.05	–0.07	–0.14	–0.30	–0.01	0.12
	(0.15)	(0.14)	(0.14)	(0.21)	(0.21)	(0.23)
Parental control X High parental occupation	0.13	–0.02	–0.06	0.00	–0.13	–0.20
	(0.16)	(0.16)	(0.14)	(0.22)	(0.16)	(0.23)
Parental warmth X Parental control X	–0.01	–0.05	–0.01	–0.13*	–0.04	–0.09
	(0.06)	(0.06)	(0.07)	(0.07)	(0.06)	(0.08)

High parental occupation						
Parental activities X High parental occupation	0.05	–0.04	0.23	–0.01	0.01	0.18
	(0.15)	(0.14)	(0.14)	(0.15)	(0.16)	(0.19)
Extracurricular activities X High parental occupation	–0.21	0.63†	0.04	–0.29	0.19	–0.10
	(0.22)	(0.35)	(0.22)	(0.42)	(0.30)	(0.39)
N (twins)	540	540	534	380	462	440

Notes: All variables (apart from high parental occupation) are z-standardized. Cluster-robust standard errors in parentheses. All models control for IQ, birth weight, academic self-concept at wave 1, intrinsic motivation at wave 1, learning motivation at wave 1, and self-efficacy at wave 1 (controls not shown).
Source: TwinLife, version 4.0.0 (doi:10.4232/1.13539).
† p < 0.10; * p < 0.05; ** p < 0.01

Table S18. Fixed-Effects Models of the Effects of Parenting on Children's Noncognitive Skills, Controlling for IQ, Birth Weight, and Noncognitive Skills at Wave 1, Dizygotic and Monozygotic Twin Panels Combined.

	(1)<br>Academic Self-Concept	(2)<br>Intrinsic Motivation	(3)<br>Learning Motivation	(4)<br>Self-Efficacy	(5)<br>Self-Esteem	(6)<br>Locus of Control
Parental warmth	0.08	0.01	0.04	0.12†	0.06	0.04
	(0.05)	(0.05)	(0.05)	(0.07)	(0.06)	(0.07)
Parental control	–0.05	–0.01	–0.01	0.12†	–0.06	–0.03
	(0.05)	(0.05)	(0.06)	(0.07)	(0.06)	(0.06)
Parental warmth	–0.02	–0.01	0.07	0.05	0.05	–0.01
X Parental control	(0.04)	(0.04)	(0.04)	(0.05)	(0.05)	(0.05)
Parental activities	–0.02	–0.03	–0.02	–0.07	–0.03	0.07
	(0.04)	(0.05)	(0.05)	(0.06)	(0.05)	(0.05)
Extracurricular	0.03	–0.05	–0.05	0.13	–0.03	0.10
activities	(0.07)	(0.09)	(0.08)	(0.10)	(0.09)	(0.09)
N (twins)	1,306	1,304	1,292	954	1,122	1,096

Notes: All variables are z-standardized. Cluster-robust standard errors in parentheses. All models control for IQ, birth weight, academic self-concept at wave 1, intrinsic motivation at wave 1, learning motivation at wave 1, and self-efficacy at wave 1 (controls not shown).
Source: TwinLife, version 4.0.0 (doi:10.4232/1.13539).† $p < 0.10$; * $p < 0.05$; ** $p < 0.01$

Table S19. Fixed-Effects Models of the Effects of Parenting on Children's Noncognitive Skills, Interaction with Child Gender, Controlling for IQ, Birth Weight, and Noncognitive Skills at Wave 1.

	(1) Academic Self-Concept	(2) Intrinsic Motivation	(3) Learning Motivation	(4) Self-Efficacy	(5) Self-Esteem	(6) Locus of Control
Panel A: Dizygotic twins						
Parental warmth	0.03	0.01	0.21*	0.21†	0.14	0.24*
	(0.10)	(0.09)	(0.09)	(0.11)	(0.09)	(0.10)
Parental control	0.01	–0.05	–0.07	0.11	–0.13	–0.08
	(0.09)	(0.08)	(0.11)	(0.09)	(0.09)	(0.10)
Parental warmth X Parental control	–0.07	0.02	0.03	0.02	0.08	–0.07
	(0.05)	(0.05)	(0.06)	(0.07)	(0.06)	(0.06)
Parental activities	0.08	0.05	0.05	–0.06	–0.08	0.05
	(0.09)	(0.08)	(0.09)	(0.10)	(0.08)	(0.09)
Extracurricular activities	0.00	0.00	–0.14	0.04	–0.09	0.04
	(0.12)	(0.13)	(0.16)	(0.17)	(0.19)	(0.17)
Parental warmth X Female	0.05	–0.15	–0.42**	–0.28	0.07	–0.30
	(0.13)	(0.12)	(0.14)	(0.19)	(0.13)	(0.20)
Parental control X Female	–0.04	0.13	0.18	–0.03	0.05	–0.04
	(0.12)	(0.12)	(0.15)	(0.20)	(0.16)	(0.15)
Parental warmth X Parental control X Female	0.00	0.02	0.09	–0.04	–0.06	–0.09
	(0.05)	(0.06)	(0.09)	(0.09)	(0.07)	(0.08)
Parental activities X Female	–0.06	–0.12	–0.05	0.05	0.03	0.05
	(0.12)	(0.12)	(0.13)	(0.16)	(0.15)	(0.13)
Extracurricular activities X Female	0.11	–0.05	0.02	0.06	0.16	0.01
	(0.17)	(0.19)	(0.21)	(0.23)	(0.23)	(0.22)
N (twins)	766	764	758	574	660	656
Panel B: Monozygotic twins						
Parental warmth	0.07	0.11	0.04	0.16	–0.08	–0.18
	(0.09)	(0.08)	(0.09)	(0.12)	(0.11)	(0.12)
Parental control	–0.09	–0.06	–0.01	0.07	0.03	0.01
	(0.11)	(0.10)	(0.10)	(0.15)	(0.11)	(0.15)
Parental warmth X Parental control	0.09	–0.11†	0.07	0.12	0.00	0.05
	(0.06)	(0.06)	(0.06)	(0.08)	(0.09)	(0.07)
Parental activities	–0.07	–0.04	0.00	–0.15	0.22†	–0.03
	(0.09)	(0.09)	(0.09)	(0.10)	(0.13)	(0.12)
Extracurricular activities	0.21	0.16	–0.05	0.09	0.13	0.23
	(0.19)	(0.18)	(0.18)	(0.26)	(0.28)	(0.21)
Parental warmth X Female	0.18	0.00	–0.05	0.14	–0.04	0.31
	(0.16)	(0.15)	(0.16)	(0.18)	(0.24)	(0.22)
Parental control X Female	–0.11	0.04	–0.05	0.27	–0.06	0.10
	(0.16)	(0.16)	(0.14)	(0.20)	(0.18)	(0.21)
Parental warmth X Parental control X Female	–0.08	–0.14†	–0.03	0.10	0.00	-0.13
	(0.05)	(0.08)	(0.04)	(0.07)	(0.07)	(0.09)
Parental activities X Female	–0.10	–0.05	–0.17	–0.06	–0.28†	0.11
	(0.13)	(0.12)	(0.13)	(0.13)	(0.16)	(0.16)
Extracurricular activities X Female	–0.42†	–0.71*	0.02	0.39	–0.20	–0.32
	(0.22)	(0.30)	(0.21)	(0.39)	(0.32)	(0.29)
N (twins)	540	540	534	380	462	440

Notes: All variables (apart from female) are z-standardized. Cluster-robust standard errors in parentheses. All models control for IQ, birth weight, academic self-concept at wave 1, intrinsic motivation at wave 1, learning motivation at wave 1, and self-efficacy at wave 1 (controls not shown).
Source: TwinLife, version 4.0.0 (doi:10.4232/1.13539).
† $p < 0.10$; * $p < 0.05$; ** $p < 0.01$
